# Clinical Scores and Machine Learning at Matched Variable Domains in Acute Appendicitis: Performance of Recommended Rule-Out Thresholds in a CT-Verified Cohort

**DOI:** 10.3390/jcm15208141

**Published:** 2026-10-20

**Authors:** Burak Celik, Ipek Pehlivan, Alper Hatipoglu, Zeynep Ece Dimitoka, Safa Toprak, Ibrahim Halil Ozata, Emre Balik, Metin Turkay

**Affiliations:** 1 Department of General Surgery, School of Medicine, Koç University, Istanbul 34450, Türkiye; satoprak@kuh.ku.edu.tr (S.T.); iozata@kuh.ku.edu.tr (I.H.O.); ebalik@ku.edu.tr (E.B.); 2 Department of Industrial Engineering, Faculty of Engineering, Koç University, Istanbul 34450, Türkiye; ipehlivan20@ku.edu.tr (I.P.); mturkay@ku.edu.tr (M.T.); 3 School of Medicine, Koç University, Istanbul 34450, Türkiye; ahatipoglu21@ku.edu.tr (A.H.); zdimitoka21@ku.edu.tr (Z.E.D.)

**Keywords:** acute appendicitis, Alvarado score, Appendicitis Inflammatory Response score, Adult Appendicitis Score, computed tomography, clinical prediction model, machine learning, emergency department, diagnostic accuracy, risk stratification

## Abstract

**Background/Objectives**: Clinical scores are recommended for excluding acute appendicitis without imaging, but their rule-out thresholds were derived in patients selected on suspicion of appendicitis. This study tested whether these thresholds retain their reported sensitivity in patients selected by CT performance, estimated the incremental diagnostic value of successive layers of clinical information, and, as an exploratory aim, compared machine learning models with the scores. **Methods**: This single centre retrospective cohort comprised 761 emergency department patients undergoing abdominal CT for acute abdominal pain. The Alvarado score, the Appendicitis Inflammatory Response (AIR) score and the Adult Appendicitis Score (AAS) were reconstructed from data documented before CT. The reference standard was histopathology in operated patients and the index CT report with the clinical course otherwise. Models were fitted to five cumulative information layers by cross-validation, with nested algorithm selection and a temporal split. **Results**: Appendicitis was confirmed in 178 patients (23.4%). Sensitivity at the recommended thresholds was 0.680 (95% CI 0.609 to 0.751), 0.848 (0.791 to 0.899) and 0.697 (0.628 to 0.764), against published values of 0.99, 0.95 and 0.959, leaving 57, 27 and 54 confirmed cases in the low probability strata, and the shortfall was present in both temporal periods. Discrimination was preserved, with areas under the curve of 0.848, 0.868 and 0.881. Model discrimination rose from 0.874 with symptoms to 0.918 with laboratory values, with each increment conditional on the preceding layers and the pattern similar with a single algorithm held fixed. With nested selection, the model was not distinguishable from the AAS, and in the later temporal period its deferral threshold attained a sensitivity of 0.925. **Conclusions**: In a population defined by imaging rather than by clinical suspicion, the recommended rule-out thresholds did not translate. Thresholds should be re-derived and verified in the population in which imaging is to be deferred. The model analysis was exploratory and was not externally validated.

## 1. Introduction

Acute abdominal pain is among the most frequent reasons for presentation to the emergency department (ED), accounting for 7–10% of all attendances [1,2]. Acute appendicitis is the most common surgical cause of such presentations and the most frequent diagnosis in young patients admitted with an acute abdomen, with a lifetime risk of 8.6% in males and 6.7% in females [3]. No single symptom, sign or laboratory test reliably distinguishes appendicitis from the many other conditions that present with right lower quadrant pain, and the assessment therefore rests on the synthesis of clinical, laboratory and radiological information [4,5]. A missed appendicitis may progress to perforation, with mortality rising from below 0.1% in uncomplicated disease to approximately 5% once perforation has occurred [5], whereas overcalling the diagnosis exposes patients to an unnecessary operation.

Ultrasonography is the recommended first-line modality but is operator-dependent, with a reported sensitivity of 44% to 94% [6]. Computed tomography (CT) offers superior performance, with sensitivity and specificity exceeding 95%, and has consequently become the standard next step in adults in whom uncertainty persists after initial assessment [6,7]. In cohorts imaged for suspected appendicitis, the majority of patients ultimately prove not to have the disease, so a substantial share of scans is performed on patients who derive no diagnostic benefit but are exposed to radiation and contrast-related risk, with additional time and cost to the department. Selective imaging strategies, in which CT follows an inconclusive first-line assessment rather than being performed immediately, were developed for this reason and reduce CT use without loss of diagnostic yield [7]. The target is therefore not fewer diagnoses but fewer scans that were never going to change management.

The classic presentation, periumbilical pain migrating to the right lower quadrant, fever and leukocytosis, is neither universally present nor specific, leaving diagnostic uncertainty in a substantial proportion of patients [4]. Clinical scores were developed to structure this uncertainty before imaging, and current guidelines recommend risk stratification as the basis for deciding who requires a scan, endorsing the Alvarado score, the Appendicitis Inflammatory Response (AIR) score and the Adult Appendicitis Score (AAS) as tools to identify low-risk patients in whom imaging may be deferred [5,8,9,10]. Each score assigns points to symptoms, examination findings and inflammatory markers and classifies patients into low, intermediate and high probability bands, with imaging recommended chiefly for the intermediate group. In a prospective cohort of 829 adults presenting with suspected appendicitis, of whom 725 had complete data for the score comparison, these low-risk thresholds excluded appendicitis with sensitivities of 0.980 for Alvarado at ≤4, 0.831 for AIR at ≤4, and 0.959 for AAS below 11 [10]. Critically, these scores were derived and validated in patients entering on clinical suspicion of appendicitis or right lower quadrant pain. Their rule-out thresholds are nevertheless applied at the point where imaging is decided, among patients with acute abdominal pain in whom the diagnosis is not yet established. Whether a threshold derived in the former population retains its sensitivity in the latter is a question of transportability, distinct from the discrimination of the score itself, and has not been characterised.

In emergency medicine, machine learning tools are increasingly explored as adjuncts to conventional diagnostic pathways, with the potential to reduce diagnostic delays, minimise unnecessary interventions and improve outcomes [11]. In appendicitis, models built on clinical, laboratory and imaging variables have reported high discriminative accuracy, but they have been developed in undifferentiated emergency populations [12], in patients already selected for equivocal imaging [13], or in operated cohorts where the outcome was appendicitis severity rather than the diagnosis itself [14], and none has been evaluated as a tool for deciding whether imaging is required.

This study addressed three questions. The primary question was whether the recommended rule-out thresholds of the Alvarado score, the AIR score and the AAS retain their reported sensitivity in a cohort defined by CT performance rather than by clinical suspicion, with the hypothesis that they do not. The secondary question was the incremental diagnostic value of symptoms, physical examination, laboratory tests and CT, estimated as successive information layers along the diagnostic pathway. The third, exploratory question was whether machine learning models fitted at matched variable domains, defined here as the categories of pre-imaging information the scores draw on, namely symptoms, examination findings and laboratory results, without implying identical information content, discriminate better than the scores.

## 2. Materials and Methods

### 2.1. Study Design and Setting

This was a single-centre retrospective cohort study conducted at Koç University Hospital, a tertiary referral centre, between November 2015 and April 2026. The protocol was approved by the Koç University Institutional Review Board (2026.255.IRB2.136). All patients provided written general consent for the use of their clinical data at the time of emergency department registration, and this consent covered the present analysis. Reporting follows the TRIPOD+AI statement [15].

### 2.2. Participants

Patients aged 16 years or older presenting to the emergency department with acute abdominal pain between November 2015 and April 2026 were screened (n = 1094). Patients were eligible if abdominal CT had been performed during the index visit. The decision to obtain CT was made by the treating emergency physician as part of routine care. No institutional protocol mandated imaging for a specific clinical presentation, symptom pattern, or clinical score value, and imaging was not restricted to patients in whom appendicitis was suspected. Patients were excluded if CT was absent or technically incomplete, if laboratory data required for score computation were incomplete, if appendectomy had been performed previously, or in the presence of confirmed or suspected pregnancy. A total of 333 patients were excluded on these grounds, and the final analysis cohort comprised 761 patients. The 333 excluded patients were removed at screening on eligibility grounds, before data extraction, so their clinical characteristics were not collected and a comparison with the analysed cohort is not possible.

CT was required for inclusion because the sequential model design incorporates structured CT findings and radiologist interpretation as information layers. Inclusion of all imaged patients, rather than those with suspected appendicitis alone, was also intended to capture the full range of diagnoses underlying acute abdominal pain. Patients were therefore not preselected on the basis of right lower quadrant pain, suspected appendicitis, or surgical consultation. This differs structurally from the cohorts in which the recommended rule-out thresholds of the Alvarado score, the AIR score, and the AAS were derived, in which right lower quadrant pain or suspected appendicitis was an entry criterion. Because imaging was available in every patient, the reference standard was obtained independently of the score values, so patients with low scores were not selectively discharged without verification.

### 2.3. Reference Standard and Outcome Definition

**Figure 1:**
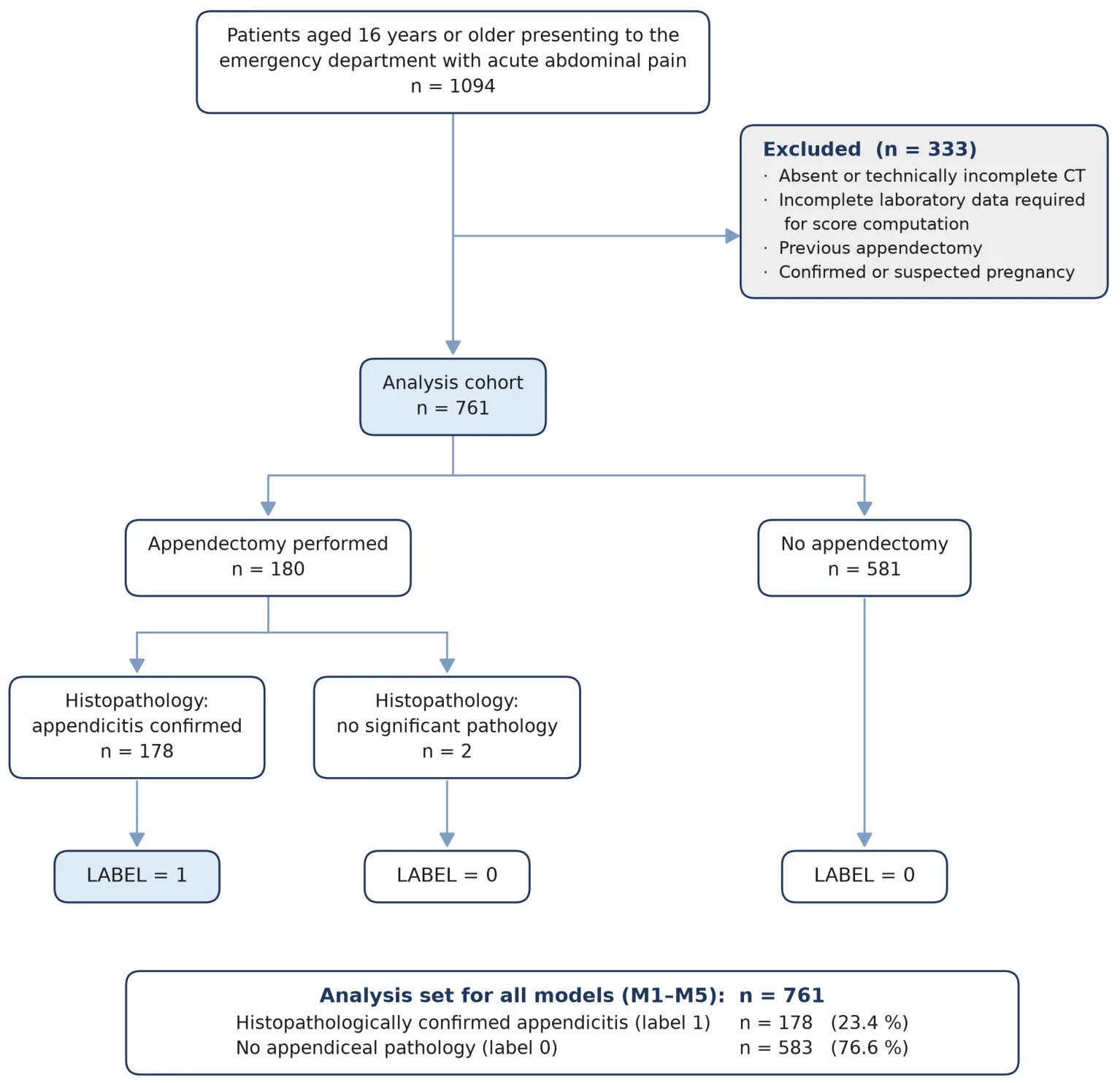
Study flow.

Among the 761 eligible patients, appendectomy was performed in 180. Histopathological examination confirmed appendicitis in 178 of these patients (98.9%); in the remaining 2 (1.1%) the resected specimen showed a faecalith without inflammatory change, representing negative appendectomy, and these patients were classified as appendicitis-negative under the reference standard. All models and all analyses of the clinical scores were developed and evaluated using the 178 histopathologically confirmed patients as the positive class (Figure 1).

The negative class comprised 583 patients: the 2 with a negative appendectomy described above, and 581 who did not undergo operation. In these 581, the outcome was classified as negative on the basis of the final diagnosis established by the treating clinician. These patients were either admitted for treatment or discharged according to that diagnosis, and the outcome classification was confirmed by structured chart review. Negative cases comprised nonspecific abdominal pain and alternative diagnoses established by operative findings, follow-up, or pathology.

The reference standard therefore differed according to outcome status. Appendicitis was confirmed by histopathological examination of the appendectomy specimen, whereas absence of appendicitis was established by the index CT report together with the documented clinical course of the presentation, in patients who did not undergo appendectomy and for whom no structured follow-up window was available. Verification was therefore differential rather than uniform, and patients classified as free of appendicitis were verified by a less definitive test than those classified as having it. Because that test is the index CT, the structured CT findings entering M4 and the radiologist interpretation entering M5 are not independent of the reference standard, and discrimination at those two layers is subject to incorporation bias.

### 2.4. Variables and Data Collection

For each patient, the following variables were extracted from the electronic medical record. Demographic characteristics comprised age and sex. Symptom-related and bedside clinical variables comprised nausea, vomiting, anorexia, duration of symptoms prior to presentation, pain migration, pain localisation, body temperature, and fever. Laboratory parameters comprised white blood cell count, leukocytosis, neutrophil and lymphocyte counts and percentages, C reactive protein, haemoglobin, potassium, and sodium. For model development, the physical examination layer was represented as a single binary composite variable indicating the presence of right lower quadrant tenderness, guarding, or rebound tenderness, reflecting routine emergency department documentation rather than separate modelling of each examination component.

Structured CT findings comprised appendiceal diameter, appendiceal wall enhancement, periappendiceal fat stranding, intra-abdominal free air, free fluid, and mesenteric lymphadenopathy, together with the final radiologist interpretation. Also, the findings were scored by a surgeon who was blinded to histopathology and who worked independently of the narrative radiology report from which the final interpretation was derived.

### 2.5. Clinical Scores

The Alvarado score, the AIR score, and the AAS were computed for every patient in the analysis set, irrespective of whether appendicitis was suspected at presentation, using only the clinical, examination, and laboratory data available before CT was performed. Score components were not recorded prospectively and were reconstructed retrospectively from the documented record. Appendicitis status was determined by the reference standard defined above.

Right lower quadrant tenderness, rebound tenderness, and guarding were documented in the record as a single composite binary finding, without the severity grading required by the AIR score and the AAS. Where this finding was positive, the highest valid grade of each instrument was assigned, namely strong rebound tenderness or muscular defence for the AIR score and moderate or severe guarding for the AAS. The Alvarado score has no graded examination item; its two examination items, right lower quadrant tenderness and rebound tenderness, were both assigned from the same composite finding. This is the assignment most favourable to the clinical scores, since a lower grade would lower scores, enlarge the low probability stratum, and increase the number of missed cases. The full range of grade assignments is reported in Supplementary Table S1.

Each score was interpreted according to the risk strata defined in its source literature, and these strata are referred to throughout as the recommended rule-out thresholds. CT deferral denotes the decision to withhold imaging below a threshold fitted at a stated sensitivity, applied to the models and to the score thresholds re-derived in this cohort. For the Alvarado score, a value of 4 or below indicates low probability of appendicitis, 5 to 6 indicates intermediate probability, and 7 or above indicates high probability [8]. For the AIR score, the original low probability stratum is 4 or below and the most recent meta-analysis reports the stratum of 3 or below [16]. Three or below is applied in the main analysis because it is the more conservative comparator, the shortfall against its pooled sensitivity being smaller than at 4 or below; results at 4 or below are reported in Supplementary Table S2. Intermediate probability is 4 to 8, and high probability is 9 or above. For the AAS, a value below 11 indicates low probability, 11 to 15 indicates intermediate probability, and 16 or above indicates high probability [10].

The lowest stratum of each score defines its recommended rule-out threshold, and every patient was classified as either falling within or above this stratum. Patients within the low probability stratum were the group in whom the score would have indicated that appendicitis could be excluded without imaging. Within this group, the number of patients with histopathologically confirmed appendicitis was determined, and these patients constitute the cases that the recommended threshold would have missed in this cohort.

For each score, sensitivity, specificity, and negative predictive value (NPV) at the recommended rule-out threshold were calculated against the reference standard, with 95% confidence intervals obtained by percentile bootstrap resampling with 3000 replications. Discrimination across the full range of each score was summarised by the area under the receiver operating characteristic curve. Discrimination was additionally assessed within the low probability stratum alone, using the same score as the predictor, in order to quantify the diagnostic information discarded by dichotomisation at the recommended threshold.

Observed operating characteristics were compared with pooled estimates from the meta-analytic literature rather than from single derivation studies. For the Alvarado score at a low probability stratum of 1 to 4, the reference values were a sensitivity of 0.99 (95% CI 0.97 to 0.99) and a specificity of 0.43 (0.36 to 0.51) across 28 studies [17]. For the AIR score at 3 or below, the reference values were a sensitivity of 0.95 (0.90 to 0.97) and a specificity of 0.47 (0.42 to 0.53) across 26 studies comprising 15,699 patients [16]. The AIR sensitivity of 0.831 quoted in the Introduction is the value observed at 4 or below in the derivation cohort of the AAS [10]; the meta-analytic estimate at the threshold analysed is the benchmark used throughout. For the AAS at a value below 11, the reference value was the sensitivity of 0.959 reported in the derivation study, as no meta-analysis of the AAS was available [10]. Because estimates of diagnostic accuracy depend on the prevalence and clinical spectrum of the population in which they are obtained, the prevalence of appendicitis in the source populations is reported alongside that of the present cohort. The pretest probability of appendicitis was approximately 66% in the adult studies of one review [18] and ranged from 32% to 91% across the studies of the other [17]; in the derivation cohort of the AAS, it was 47%, with 392 of 829 enrolled patients and 343 of the 725 with complete data used for the score comparison [10]. The corresponding figure in the present cohort is 23.4%.

### 2.6. Machine Learning Models

**Figure 2:**
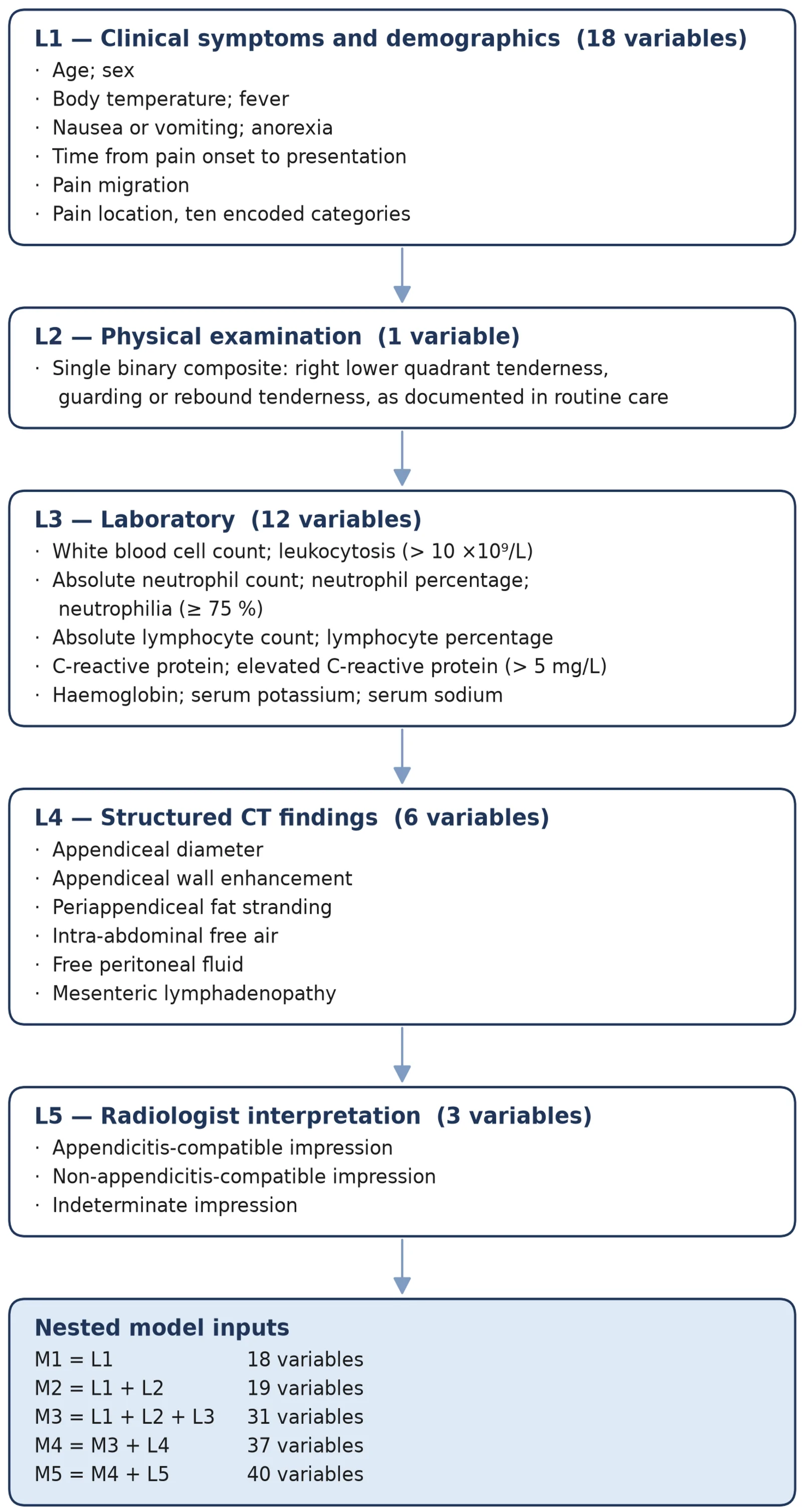
Variable taxonomy across diagnostic information layers.

Predictors were organized into five sequential information layers (L1–L5), reflecting the order in which clinical data become available: patient-reported symptoms and demographics (L1), physical examination findings (L2), routine laboratory results (L3), structured CT findings (L4), and the radiologist’s final CT interpretation (L5). From these layers, five cumulative feature sets were constructed (M1–M5), each incorporating all information available up to that point, so that M1 used L1 only and each subsequent model added the next layer (Figure 2). This allowed performance to be evaluated as information accumulates along the clinical pathway rather than as a single static prediction problem.

Three established clinical scoring systems were included as reference benchmarks. All three combine symptoms, examination findings and laboratory results, so the matched variable domain for each is M3, and this is the primary comparison [8,10,19]; comparisons against M1 and M2 are additionally reported as a conservative contrast in which the model has access to less information than the score. M3 also contains serum sodium, potassium and haemoglobin, which none of the scores use, and uses the continuous rather than the dichotomised form of the shared laboratory variables, so the comparison is matched on variable domain rather than on information content.

Eleven machine learning algorithms were evaluated, comprising regularized linear models (logistic regression, ElasticNet, and ridge), a support vector machine with a radial basis function kernel, tree ensembles (random forest and extra trees), and gradient-boosted decision trees (gradient boosting, HistGradientBoosting, XGBoost, LightGBM, and CatBoost). Hyperparameters were fixed a priori rather than tuned by grid search, reducing optimism in a moderate-sized cohort; the values used are given in Supplementary Table S3. Deep learning was not used, given the sample size and tabular structure, for which tree-based and linear methods perform more reliably. Relative to the 178 outcome events, the candidate predictors correspond to 9.9 events per variable at M1, 9.4 at M2, 5.7 at M3, 4.8 at M4 and 4.5 at M5. The sample size was fixed by data availability rather than by a power calculation, and the lower ratios at the CT-inclusive layers are acknowledged in the limitations. Complete data for every modelled variable were required for eligibility, so no imputation was performed; class imbalance was addressed through model-level class weighting, without synthetic oversampling, which can distort probability estimates in smaller datasets.

Unless stated otherwise, all results reported below are based on 10-fold stratified cross-validation over the whole cohort (n = 761). Every patient contributes one out-of-fold prediction from a model fitted to the remaining nine folds. Within each layer, the algorithm with the highest cross-validated AUC was carried forward, and the CT-deferral threshold was likewise derived within each fold from the remaining nine. Because reporting the best of eleven cross-validated estimates is optimistic, the whole procedure was repeated with the selection nested inside the cross-validation. Within each outer fold, the eleven algorithms were compared by 10-fold cross-validation on the training part only, the winner was refitted on that part and applied to the outer fold, and the deferral threshold was set from the winner’s inner out-of-fold predictions by the same rule (Supplementary Table S4). Two sensitivity targets were specified in advance and applied unchanged to the models and to the clinical scores. No guideline states an acceptable miss rate for appendicitis; the clinical target for deferring CT was set a priori at 0.95 or above, corresponding to the pooled sensitivity reported for the AIR score at its recommended threshold. Thresholds were fitted at the stricter target of 0.97 or above, with a margin of 0.02 intended to prevent the sensitivity attained outside the fold from falling below the clinical target once the threshold was transferred. Because the scores and the model outputs take discrete values, neither target can be attained exactly; the threshold applied was the highest value whose sensitivity in the remaining nine folds was at or above 0.97, that is, the attainable operating point closest to the fitting target from above. No patient contributed to the threshold applied to them, and the same fold-external procedure was applied to the clinical scores wherever a matched safety target is reported. The sensitivity attained out of fold is reported with its confidence interval alongside every deferral result; these are Wilson intervals, with the percentile bootstrap being unreliable with as few as two to six missed cases.

Cross-validation rather than a single split was used as the primary internal validation because split-sample validation is inefficient at this sample size, fitting the model on fewer patients and estimating its performance on fewer events [20]. The cohort was additionally partitioned by stratified random sampling into training (n = 456, 107 with appendicitis), validation (n = 152, 35) and held-out test (n = 153, 36) subsets. Models were fitted on the training set, the Youden J operating threshold and the recalibration map were selected on the validation set, and performance was measured once on the held-out test set; the algorithm carried forward at each layer was selected within that partition and therefore differs from the cross-validated selection at four of the five layers. These results are reported in Supplementary Table S5. Because the 153 held-out patients are a subset of the 761, the two analyses are not independent, and the held-out estimates are not a substitute for external validation. The cross-validated AUC of every algorithm at every layer is reported in Supplementary Table S7. Because the algorithm carried forward changed between layers, the layer analysis was repeated with a single algorithm, ridge logistic regression with its prespecified hyperparameters (Supplementary Table S3), held fixed at every layer; with the same algorithm, each pre-imaging layer was also evaluated alone, and the pre-imaging layers were added in a laboratory-first order, L3, then L1, then L2 (Supplementary Table S8). For temporal validation, patients presenting before 1 January 2024 formed the development set, and those presenting from that date the test set, a split such that the test set retained enough events. In the development set, the eleven algorithms were compared at M1, M2 and M3 by 10-fold cross-validation, and the best was refitted on all development patients and applied once to the test set. Score thresholds and the M3 deferral threshold were re-derived in the development set by the rule above, for M3 on its development out-of-fold predictions, and applied unchanged to the test set Model stability was additionally checked for M3 to M5 with 20 repetitions of 5-fold cross-validation. Model development and reporting followed the TRIPOD+AI guidance [15] (Supplementary Figure S1).

### 2.7. Statistical Analysis

Model performance was evaluated using the area under the receiver operating characteristic curve (AUC), sensitivity, specificity, positive predictive value, NPV, and the Brier score [21]. Calibration was assessed on the out-of-fold predictions using the Brier score, the calibration slope—the coefficient of logit($\hat{p}$) in a logistic regression of the observed outcome on the linear predictor, where 1 indicates perfect calibration—and the calibration intercept, obtained from the same model with the slope fixed at 1, where 0 indicates perfect mean calibration [22,23]. Confidence intervals for the slope and intercept are Wald intervals from the logistic fit; those for the Brier score are percentile intervals from 3000 patient-level bootstrap resamples. Calibration curves were drawn by locally weighted scatterplot smoothing of the observed outcome on the predicted probability, with observed proportions within deciles of predicted risk [23]. The scaled Brier score, 1 − Brier/[$\bar{p}$(1 − $\bar{p}$)], compares the Brier score with that of a model assigning the observed prevalence $\bar{p}$ to every patient, for which $\bar{p}$(1 − $\bar{p}$) = 0.1792; 1 indicates perfect prediction and 0 no improvement over that model. The Hosmer–Lemeshow test was not used; because its result depends on arbitrary grouping, it does not indicate the direction of miscalibration, and its power increases with sample size [24,25]. Sequential transitions between cumulative feature sets (M1→M2, M2→M3, M3→M4, and M4→M5) were treated as prespecified contrasts, as were the comparisons between each model and the clinical scores. Because the out-of-fold prediction for a given layer is produced by ten separately fitted models, and the DeLong test for paired AUC curves treats the classifiers as fixed and therefore does not propagate the variability of the fitting procedure, the primary analysis for both sets of contrasts was a patient-level bootstrap of 300 resamples in which every model was refitted within the resample and evaluated on the patients left out of it, with the clinical scores evaluated in the same left-out patients so that each contrast is paired. Within each resample, the algorithm carried forward at each layer was refitted; for the contrasts of M3 against the scores, the bootstrap was repeated with 1000 resamples in which the selection among the eleven algorithms was itself repeated within each resample, by cross-validation on the resample grouped by patient, so that those intervals include the optimism of the selection. The number of resamples was set at 300 because every model at every layer is refitted within each one, and the confidence intervals rather than the *p* values are therefore the primary summary. Two-sided *p* values are twice the smaller proportion of resamples in which the difference did not exceed zero; thus, 0.007 is the smallest value 300 resamples can resolve, and contrasts reaching that floor are reported as below 0.01. DeLong tests are reported alongside for comparability with the existing literature. Heterogeneity between sexes was tested with Fisher’s exact test where any cell count was below five and by the chi-square test with continuity correction otherwise, and differences in the area under the curve were tested with a z-test on DeLong variances for independent samples; these comparisons were exploratory.

## 3. Results

### 3.1. Cohort Characteristics and Clinical Benchmarks

A total of 761 patients were included in the analytic cohort, and their demographic, clinical, laboratory and imaging characteristics are summarised in Table 1. Appendectomy was performed in 180 patients (23.7%). Histopathological examination confirmed appendicitis in 178 of these patients (98.9% of operated patients), whereas appendicitis was excluded in two patients (1.1%), who were recorded as negative appendectomies and classified as appendicitis-negative under the reference standard. The final histopathologically verified appendicitis prevalence in the cohort was 23.4% (178/761).

Alvarado, AIR and AAS scores were reconstructed for all 761 patients from information documented before CT. Observed values covered the entire theoretical range of the Alvarado score and the AAS, and all but the highest point of the AIR score (Alvarado 0 to 10, AIR 0 to 11, AAS 0 to 23). Median scores were low across the cohort (Alvarado 3, IQR 1 to 5; AIR 3, IQR 1 to 5; AAS 6, IQR 3 to 10), reflecting a population defined by CT performance rather than by clinical suspicion of appendicitis (Table 2A).

All three instruments separated confirmed appendicitis from non-appendicitis patients. Median scores in patients with appendicitis versus without appendicitis were six (IQR 4 to 7) versus two (IQR 0 to 4) for Alvarado, six (IQR 5 to 8) versus two (IQR 1 to 4) for AIR, and 14 (IQR 9 to 17) versus five (IQR 3 to 7) for AAS.

The AUC was 0.848 (95% CI 0.815 to 0.881) for Alvarado, 0.868 (0.837 to 0.896) for AIR and 0.881 (0.850 to 0.910) for AAS across the full cohort (Table 2A). Restricting the analysis to patients below each rule-out threshold, the scores retained discriminative information that the dichotomised threshold discards. The AUC within the low-risk band was 0.697 (0.637 to 0.757) for Alvarado across scores 0 to 4, 0.726 (0.652 to 0.795) for AIR across scores 0 to 3, and 0.739 (0.676 to 0.801) for AAS across scores 0 to 10 (Table 2B). Sensitivity at the recommended thresholds under all alternative grade assignments is reported in Supplementary Table S1, and performance of the AIR score at its original threshold of four or below in Supplementary Table S2.

The sensitivity shortfall was present in both sexes and was not concentrated in women. Appendicitis prevalence was 17.9% in women (77 of 430) and 30.5% in men (101 of 331). Sensitivity of the Alvarado threshold was 0.727 (95% CI 0.623 to 0.824) in women against 0.644 (0.549 to 0.736) in men, and sensitivity of the AAS threshold was 0.675 (0.571 to 0.776) against 0.713 (0.620 to 0.803), while the AIR threshold performed similarly in both, at 0.831 (0.746 to 0.909) and 0.861 (0.792 to 0.925) (Table 3). Specificity of the Alvarado threshold was 0.841 in women against 0.909 in men. The difference between sexes was not significant for any instrument (*p* = 0.306, 0.729 and 0.707 for sensitivity) or for the M3 deferral rule, which missed three cases in each sex. Specificity of the Alvarado threshold was lower in women (*p* = 0.026), as the source meta-analysis reports, but not after correction for multiple comparisons (*p* = 0.210), and discrimination did not differ by sex for any instrument or for M3.

Of the 178 confirmed cases, 42 had none of the three localizing features on which the scores depend, with no right lower quadrant pain, no documented tenderness, guarding or rebound, and no pain migration. These patients presented earlier than the remaining confirmed cases, with a median symptom duration of 12 h against 24 h (*p* = 0.022) and with a lower inflammatory response, with a median CRP of 7.9 mg/L against 21.4 mg/L (*p* = 0.009). Body temperature was lower (*p* = 0.006) and a temperature of 37.3 °C or above less frequent (*p* = 0.021) in the same group. Of the 57, 27 and 54 confirmed cases inside the low probability strata of the Alvarado score, the AIR score and the AAS, 33, 25 and 42, respectively, were among these 42. Appendiceal diameter, wall enhancement, periappendiceal fat stranding and the radiologist’s interpretation did not differ between the two groups, and all six cases missed by the M3 deferral rule were among these 42 patients (Table 4).

### 3.2. Sequential Diagnostic Performance

**Figure 3:**
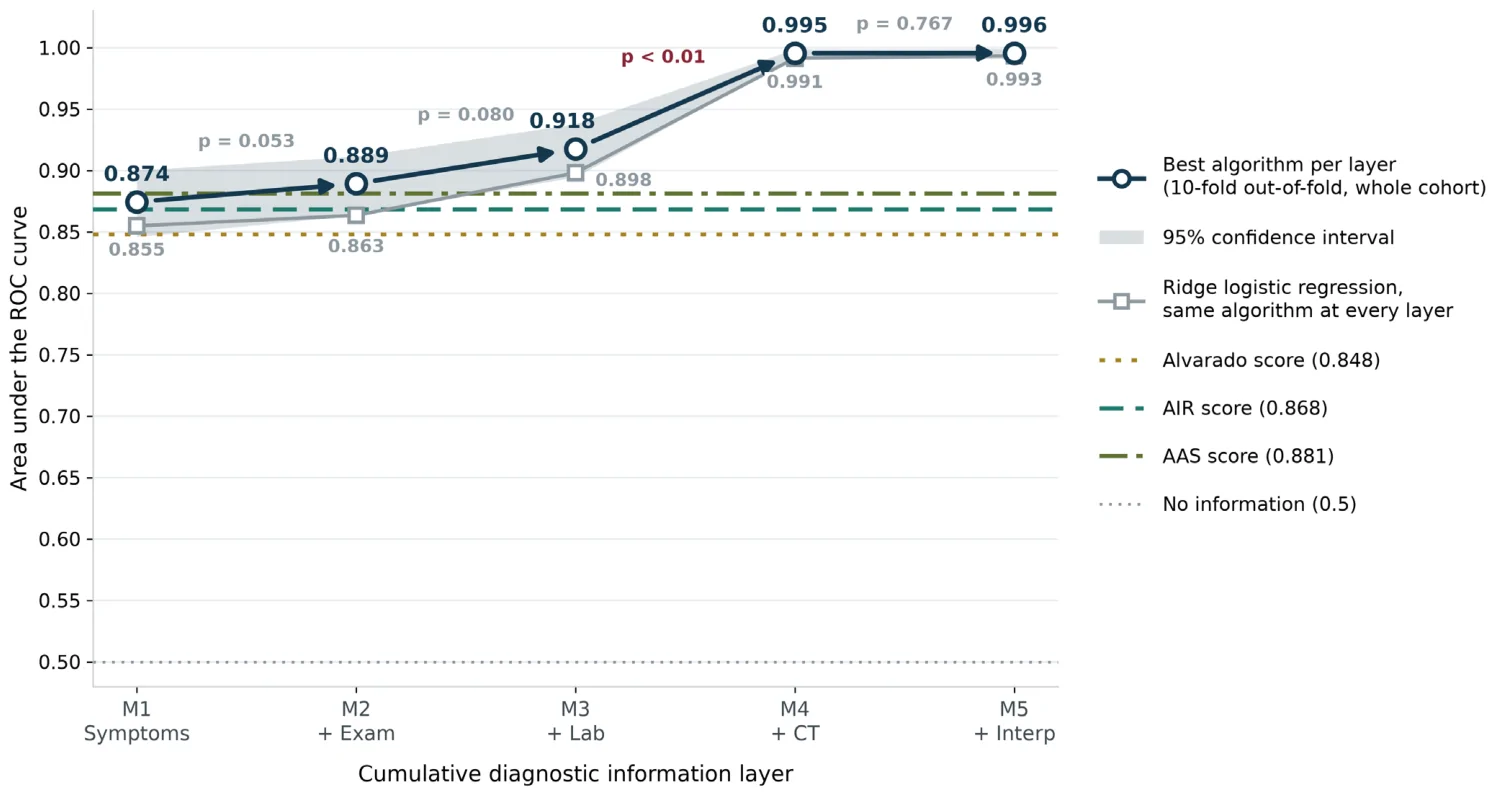
Sequential diagnostic performance across information layers.

Predictive performance increased across the information layers, although with the best algorithm at each layer only the step from M3 to M4 was statistically robust. Each increment is conditional on the layers that precede it and does not measure the value of a layer in isolation. The symptom-only model (M1) achieved a cross-validated AUC of 0.874 (95% CI, 0.846 to 0.900), rising to 0.889 (0.864 to 0.911) with physical examination (M2) and 0.918 (0.895 to 0.938) with laboratory values (M3). The algorithm carried forward was random forest at M1, M2 and M3, so the pre-imaging increments were estimated with the same algorithm, and gradient boosting at M4 and XGBoost at M5 (Supplementary Table S7); the mean AUC of the eleven algorithms was 0.860, 0.872, 0.905, 0.992 and 0.993 at M1 to M5. Increments between adjacent layers were estimated by a patient-level bootstrap in which every model was refitted within each of 300 resamples, so that the variability of the fitting procedure is propagated alongside sampling variability. Because every layer is re-estimated inside each resample, these increments are not the arithmetic differences of the out-of-fold estimates above. The increment was +0.017 (95% CI −0.001 to +0.032; *p* = 0.053) for M1 to M2 and +0.029 (−0.004 to +0.059; *p* = 0.080) for M2 to M3, neither distinguishable from zero. The addition of structured CT findings (M4) produced the only unambiguous step change, to 0.995 (0.991 to 0.999), an increment of +0.082 (+0.054 to +0.116; *p* < 0.01) that exceeded the two pre-imaging increments combined. The structured CT variables are the radiological criteria by which appendicitis is diagnosed, so this layer marks the ceiling imposed by the reference standard rather than the discovery of an independent predictor, and the radiologist interpretation layer (M5) added nothing measurable beyond it (0.996; +0.000, −0.006 to +0.006; *p* = 0.767), as expected once discrimination has reached that ceiling (Figure 3). The corresponding DeLong tests, which treat the classifiers as fixed and therefore do not propagate the variability of the fitting procedure, gave *p* = 0.025, 0.007, <0.001 and 0.863. With ridge logistic regression held fixed at every layer, the AUC was 0.855 (95% CI 0.821 to 0.886) at M1, 0.863 (0.831 to 0.893) at M2, 0.898 (0.871 to 0.924) at M3, 0.991 (0.983 to 0.998) at M4 and 0.993 (0.985 to 0.999) at M5, and the increments were +0.009 (−0.010 to +0.021; *p* = 0.267), +0.031 (+0.004 to +0.053; *p* = 0.027), +0.096 (+0.065 to +0.132; *p* < 0.01) and +0.002 (−0.003 to +0.010; *p* = 0.333) (Figure 3, Supplementary Table S8). The step from M3 to M4 was therefore of similar size with the algorithm held fixed, +0.096 against +0.082, and does not arise from the change of algorithm; the laboratory increment, not distinguishable from zero with random forest, was distinguishable with ridge regression. Evaluated alone, the symptom layer reached an AUC of 0.855 (0.821 to 0.886), the examination finding 0.793 (0.757 to 0.829) and the laboratory layer 0.783 (0.745 to 0.820). With laboratory values entered first, the AUC rose to 0.890 (0.862 to 0.917) when symptoms were added and to 0.898 when the examination finding was added, so the gain attributed to a layer depends on the layers that precede it.

### 3.3. Comparison with Clinical Scoring Benchmarks

Across the same patients, the clinical scores achieved AUCs of 0.848 (95% CI, 0.815 to 0.881) for Alvarado, 0.868 (0.837 to 0.896) for AIR, and 0.881 (0.850 to 0.910) for AAS. Model-versus-score contrasts were estimated by the same patient-level bootstrap used for the sequential increments, with the model being refitted within each resample and both the model and the score evaluated in the patients left out of it. At the matched variable domain, with the algorithm fixed as in the primary analysis, M3 discriminated better than all three: +0.065 (95% CI +0.036 to +0.093) against the Alvarado score, +0.044 (+0.015 to +0.074) against the AIR score and +0.032 (+0.003 to +0.060) against the AAS, with every interval excluding zero (*p* < 0.01, *p* < 0.01 and *p* = 0.033). The corresponding DeLong estimates were larger with smaller *p* values, at +0.070, +0.049 and +0.036, all *p* < 0.001, as expected of a test that holds the classifier fixed. In the conservative contrasts, in which the model has access to less information than the score, no difference was distinguishable from zero: M2 against the Alvarado score +0.036 (−0.003 to +0.068; *p* = 0.060), against the AIR score +0.015 (−0.020 to +0.050; *p* = 0.407) and against the AAS +0.003 (−0.033 to +0.039; *p* = 0.860), and M1 differed from none of the three. Under DeLong, the M2 to Alvarado contrast reached significance (+0.042; *p* = 0.003); it did not survive resampling of the fitting procedure. With the algorithm selection nested inside the cross-validation, the area under the curve of M3 was 0.913 (0.889 to 0.936), with random forest being selected in nine of the ten outer folds, and the nested procedure deferred CT in 302 patients (39.7%) while missing six cases, at an attained sensitivity of 0.966 (0.928 to 0.984). With the selection repeated inside every resample, the contrasts were +0.058 (+0.024 to +0.090), +0.038 (+0.006 to +0.067), and +0.025 (−0.007 to +0.052) against the Alvarado score, the AIR score and the AAS; the interval for the AAS included zero, so M3 and the AAS were not distinguishable at the matched variable domain (Supplementary Table S4).

**Figure 4:**
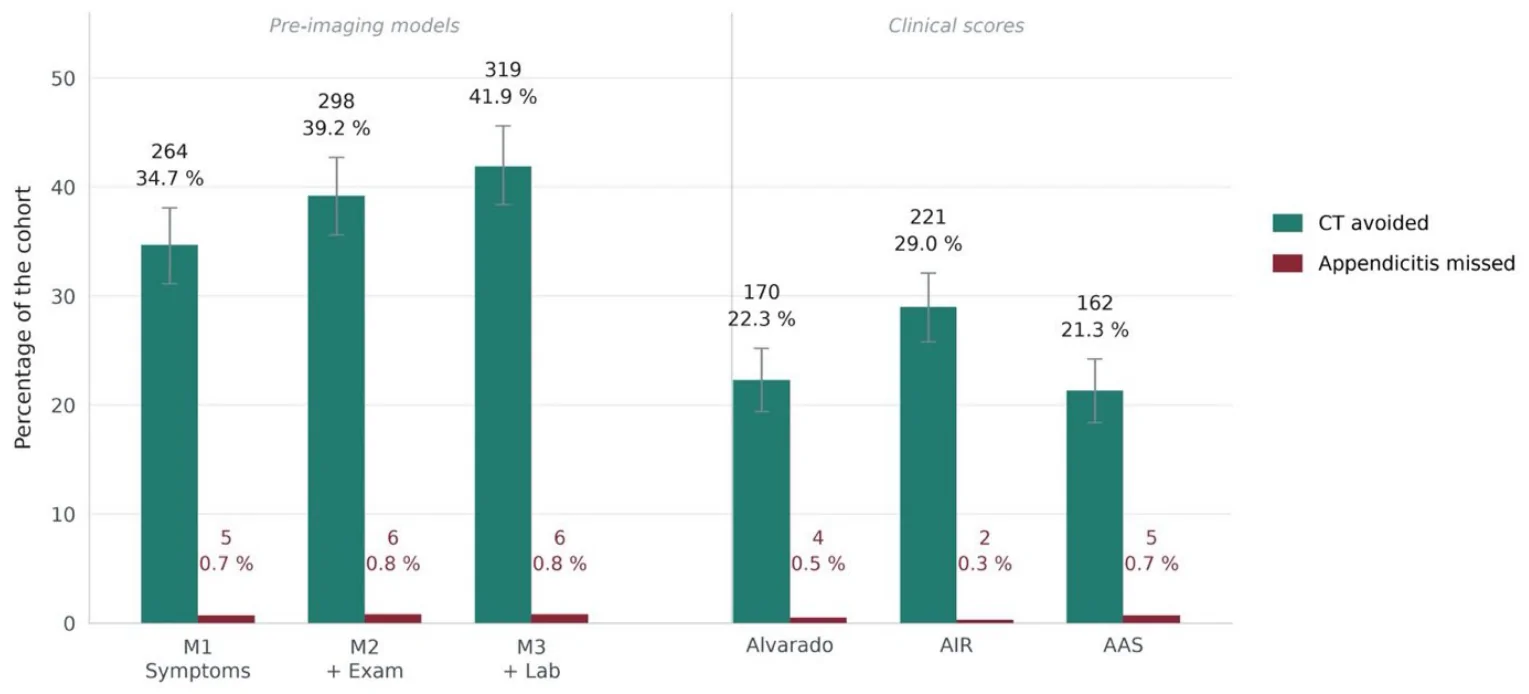
CT avoided and cases missed at a matched safety target (n = 761, 178 confirmed appendicitis).

With thresholds fitted external to the fold in which they were applied, at a fitting target of 0.97 or above against a prespecified clinical target of 0.95, M3 avoided CT in 319 of 761 patients (41.9%) while missing 6 of 178 confirmed cases, at an attained sensitivity of 0.966 (95% CI 0.928 to 0.984), M2 in 298 (39.2%) while missing 6, at 0.966 (0.928 to 0.984), and M1 in 264 (34.7%) while missing 5, at 0.972 (0.936 to 0.988). With their thresholds re-derived by the same fold-external procedure rather than taken from the literature, the AIR score avoided CT in 221 patients (29.0%) while missing 2, at 0.989 (0.960 to 0.997), the Alvarado score in 170 (22.3%) while missing 4, at 0.978 (0.944 to 0.991), and the AAS in 162 (21.3%) while missing 5, at 0.972 (0.936 to 0.988) (Figure 4). Because each predictor takes discrete values, only a finite set of operating points is attainable, and the six methods do not sit at the same sensitivity, so the numbers of examinations avoided are not directly comparable between them. The confidence intervals overlap across all six, including the widest separation, between M3 and the AIR score. The attained sensitivity of M3 was 0.966, above the prespecified clinical target of 0.95 but below the 0.97 at which its threshold was fitted, which is the loss the 0.02 margin was intended to absorb. Its lower confidence bound of 0.928 corresponds to one missed appendicitis in every 14.

**Figure 5:**
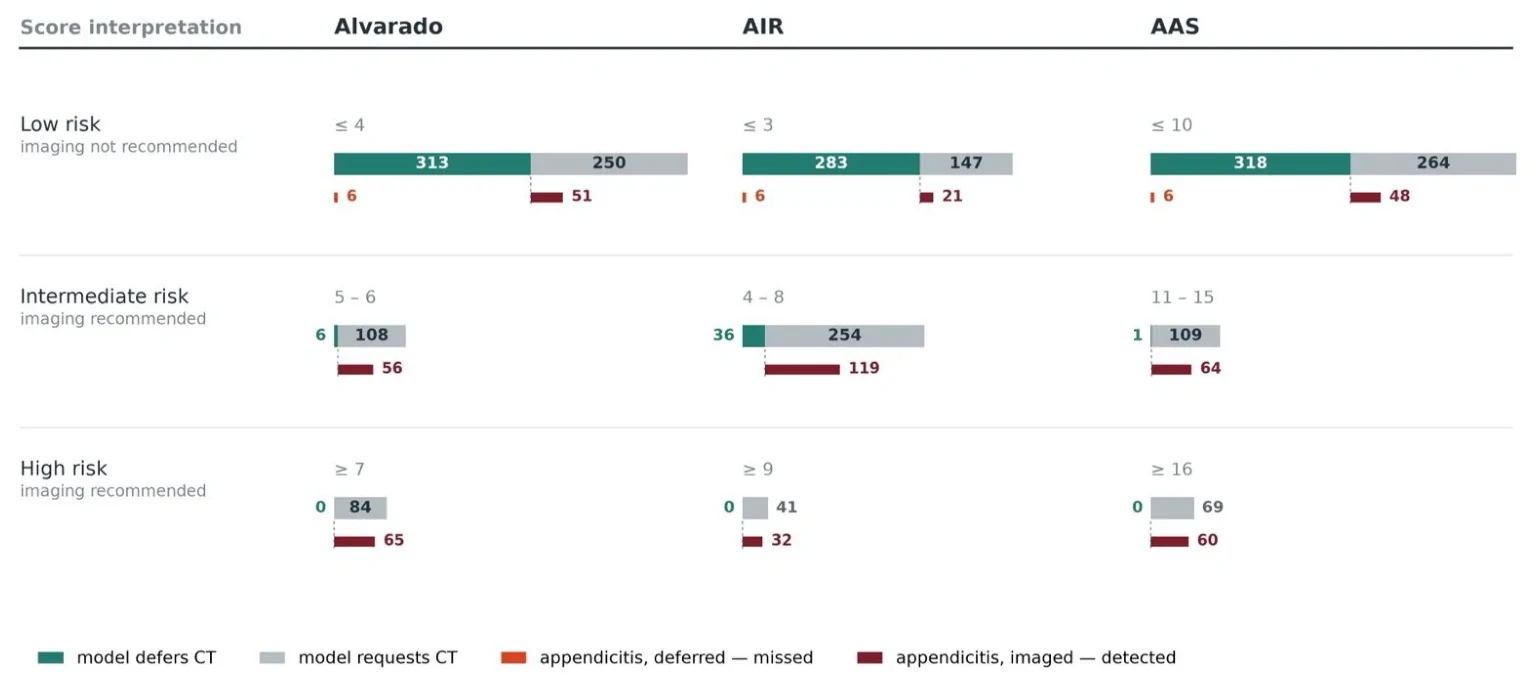
Imaging decisions within clinical-score risk bands (n = 761, 178 confirmed appendicitis).

At their recommended thresholds, the low-risk bands contained 57 confirmed cases for Alvarado, 27 for AIR, and 54 for AAS. The model retained CT in 51, 21, and 48 of these, respectively (Figure 5). The patients the model would have spared lay almost entirely inside these same bands: of the 319 deferred by M3, 313 fell in the Alvarado low-risk band, 283 in the AIR band and 318 in the AAS band, and none fell in any high-risk band. The model therefore selected the same region of the cohort as the scores rather than a different one, differing only in where it placed the boundary within that region, which at the matched safety target reported above corresponded to 319 deferrals with 6 missed cases against 162 to 221 deferrals with 2 to 5 missed cases for the scores.

### 3.4. Model Calibration and Stability

Calibration was assessed on the same out-of-fold predictions used for discrimination (761 patients, 178 with confirmed appendicitis). Brier scores fell across the information layers, from 0.121 (95% CI 0.107 to 0.134) for the symptom-only model (M1) to 0.116 (0.102 to 0.129) at M2 and 0.096 (0.084 to 0.108) at M3, and to 0.018 (0.010 to 0.026) at M4 and 0.017 (0.009 to 0.025) at M5 once CT findings entered. The corresponding scaled Brier scores were 0.327, 0.354, 0.466, 0.901 and 0.906. Calibration slopes were compatible with unity at M1 (1.09, 95% CI 0.92 to 1.26), M2 (1.08, 0.92 to 1.25) and M5 (0.93, 0.76 to 1.10). At M3 the slope was 1.27 (1.08 to 1.46), indicating predictions that were too moderate, a pattern expected of an averaged tree ensemble, and at M4 it was 0.73 (0.59 to 0.86), indicating predictions that were too extreme. Calibration intercepts were negative at every layer, indicating systematic over-prediction of risk: −0.48 (95% CI −0.69 to −0.28) at M1, −0.52 (−0.73 to −0.31) at M2, −0.25 (−0.46 to −0.04) at M3, −0.27 (−1.03 to 0.50) at M4 and −0.80 (−1.43 to −0.17) at M5; the interval excluded zero at every layer except M4. The direction of this miscalibration is the expected consequence of class weighting, which up-weights the minority class during fitting and shifts predicted probabilities upward. Calibration curves are shown in Supplementary Figure S2; at M3 the over-prediction was concentrated at predicted risks below 0.2. For M3, the pre-imaging model, the mean predicted risk was 0.263 against an observed prevalence of 0.234. Because the deferral rule applies a threshold to the ranked predictions rather than to the absolute probability, this miscalibration does not affect the CT-deferral results reported above; it bears on any use of the predicted probability as a number quoted to a patient or clinician and would require recalibration before such use. Model stability was consistent across resampling: over 20 repetitions of 5-fold cross-validation, the area under the curve was 0.918 ± 0.024 for M3, 0.995 ± 0.007 for M4 and 0.995 ± 0.007 for M5.

### 3.5. Temporal Validation

The development period, November 2015 to December 2023, included 418 patients, 111 (26.6%) with appendicitis, and the test period, January 2024 to April 2026, 343 patients, 67 (19.5%) with appendicitis. Random forest was again selected at M1, M2 and M3. In the test period the AUC was 0.836 (95% CI 0.788 to 0.880) for M1, 0.854 (0.807 to 0.896) for M2 and 0.883 (0.832 to 0.927) for M3. Calibration slopes were 0.88 (0.65 to 1.12), 0.91 (0.68 to 1.14) and 1.12 (0.85 to 1.39), each compatible with unity, and calibration intercepts were −0.99 (−1.31 to −0.67), −1.06 (−1.39 to −0.74) and −0.72 (−1.04 to −0.39), indicating over-prediction larger than in the cross-validated analysis, consistent with the lower prevalence of the test period. With thresholds re-derived in the development period and applied unchanged, M3 deferred CT in 163 of 343 test patients (47.5%) while missing 5 of 67 cases, at a sensitivity of 0.925 (0.837 to 0.968), a point estimate below the clinical target of 0.95, although its interval included it. The re-derived score thresholds, an Alvarado score of 0, an AIR score of 1 or below and an AAS of 2 or below, deferred CT in 77 (22.4%), 94 (27.4%) and 65 (19.0%) patients, each missing one case, at 0.985 (0.920 to 0.997). At the recommended thresholds, sensitivity was 0.694 (0.608 to 0.780) in the development period and 0.657 (0.537 to 0.769) in the test period for the Alvarado score, 0.838 (0.769 to 0.905) and 0.866 (0.779 to 0.940) for the AIR score, and 0.685 (0.600 to 0.769) and 0.716 (0.603 to 0.819) for the AAS, so the shortfall was present in both periods (Table 5).

### 3.6. Figures, Tables and Schemes

Predictors were grouped into five clinically ordered layers: symptoms and demographics (18 variables), physical examination (one variable), laboratory results (12), structured CT findings (six) and radiologist CT interpretation (three).

Area under the ROC curve for the best cross-validated model at each cumulative feature set, from symptoms and demographics (M1) to the full model including radiologist interpretation (M5), estimated out-of-fold over all 761 patients. The grey line shows ridge logistic regression held fixed at every layer, and the dotted line at 0.5 marks no discrimination; *p* values are for the increments of the best-algorithm line between adjacent layers, from the patient-level bootstrap with refitting. The increase at M4 and M5 reflects CT findings that also determine the reference standard in non-operated patients and is not evidence of model superiority.

Thresholds were fitted within each cross-validation fold using the remaining nine folds, at a fitting target of 0.97 or above against a prespecified clinical target of 0.95, and were applied to the clinical scores as well as to the models rather than the recommended cut-offs. Counts are shown above each bar; error bars on the CT-avoided bars are 95% percentile bootstrap intervals from 3000 resamples. Confidence intervals for the attained sensitivity, reported in the text, are Wilson intervals.

Predictions are from the pre-imaging model (M3), out-of-fold, with the decision threshold fitted within each cross-validation fold using the remaining nine folds at a fitting target of 0.97 or above, against a prespecified clinical target of 0.95.

Upper bars show all patients in the band, split by the model’s decision; lower bars, on the same scale, show patients with confirmed appendicitis aligned beneath the segment they fall in.

The six deferred cases are the same six patients in all three panels, because the model’s decision does not depend on the score bands.

Sensitivity and specificity are calculated at the recommended rule-out threshold of each instrument within each sex stratum: 95% confidence intervals from percentile bootstrap resampling with 3000 replications within the stratum, and Wilson intervals for the sensitivity of M3. P is used for tests of heterogeneity between sexes, Fisher’s exact test where any cell count was below five, chi-square test with continuity correction, and otherwise unadjusted. After Holm correction across the eight tests, the *p* value for the specificity of the Alvarado threshold was 0.210, and all others were 1.000. Area under the curve, women against men: Alvarado 0.845 against 0.858 (*p* = 0.704); AIR 0.855 against 0.882 (*p* = 0.397); AAS 0.857 against 0.897 (*p* = 0.198); M3 0.899 against 0.930 (*p* = 0.187); z-test on DeLong variances.

## 4. Discussion

### 4.1. Principal Findings

In suspected appendicitis, diagnostic value lies not only in which variables are measured, but in when they become available and what clinical action they can safely support. Prediction models in appendicitis have typically been evaluated as classifiers against a reference standard rather than as an aid to the imaging decision itself. Where the diagnostic pathway has been modelled in stages, as by Schipper et al. up to examination and again with laboratory results (areas under the curve of 0.919 and 0.923 against 0.824 for the Alvarado score) [12], the yield of examinations that could be safely withheld at a stated target has not been reported. Modelling the diagnostic pathway as sequential information layers and applying the recommended rule-out thresholds of three established scores to a cohort in which imaging was performed and the outcome ascertained in every patient separates two questions that are usually answered together, namely how much a given layer of information can discriminate and at what point in the pathway a decision based on it becomes safe.

Applied unchanged, the recommended rule-out thresholds fell 10 to 31 percentage points below the sensitivities reported in the source literature: 0.680 against a pooled 0.99 for the Alvarado score [17], 0.848 against a pooled 0.95 for the AIR score at the same threshold of three or below [16], and 0.697 against 0.959 in the derivation cohort of the AAS [10]. The low-risk band contained 57 confirmed cases for the Alvarado score, 27 for the AIR score, and 54 for the AAS, out of 178 confirmed cases. Because CT was obtained in every patient before the scores were reconstructed, these are direct counts of the cases each threshold would have discharged rather than estimates conditioned on a subsequent clinical decision. The size of each gap depends on the comparator: for the AIR score the shortfall is 10 percentage points against the pooled 0.95 at three or below, and 15 points against the pooled 0.91 at its original threshold of four or below (Supplementary Table S2).

### 4.2. Why the Thresholds Did Not Transport

The shortfall was specific to the threshold rather than to the instruments themselves. Discrimination across the full range of each instrument was at least as good as the published estimates: 0.868 for the AIR score against a pooled 0.86 [16], 0.881 for the AAS against 0.882 in its derivation cohort [10], and 0.848 for the Alvarado score against a pooled 0.79 [16], which is higher rather than lower. The grading assumption applied to the ungraded examination finding was the one most favorable to the scores.

The 42 confirmed cases carried none of the localizing features on which the scores depend presented earlier and with a lower inflammatory response than the remaining cases. The pattern corresponds to the natural history of appendicitis, in which visceral epigastric or periumbilical pain precedes somatic localization, and the inflammatory response develops over time. These patients are not a marginal subgroup, since they account for 23.6% of the confirmed cases in this cohort.

Two features of the design bear on this group. Patients judged to be at low risk and discharged without imaging are absent from the cohort, so the confirmed cases are those in whom diagnostic uncertainty persisted after initial assessment, a spectrum that may be weighted towards early and atypical presentations, in which the scores are least sensitive. The shortfall therefore describes the population in which CT is considered rather than all patients presenting with abdominal pain. Score components were also reconstructed from the record, and a localizing finding that was present but not documented would have been counted as absent, which cannot be excluded among the 42 cases. Undocumented findings cannot, however, account for the whole shortfall. Among the 136 confirmed cases with a documented localizing feature, the recommended thresholds still left 24, 2 and 12 cases inside the low probability strata of the Alvarado score, the AIR score and the AAS (Table 4) and the 42 cases presented earlier and with a lower inflammatory response, representing the pattern expected of pain that has not yet localized rather than of incomplete documentation alone.

The three scores were derived and validated in populations entering on suspicion of appendicitis or right lower quadrant pain, with a pretest probability of approximately 66% in the adult studies of one review [18] and a range of 32% to 91% across the studies of another [17], against 23.4% here. A patient whose pain has not yet localized does not meet those entry criteria. Partial verification bias offers a weaker account, since one of these reviews restricted inclusion to prospective cohort studies with follow-up of non-operated patients and still reported a negative likelihood ratio of 0.03 for an Alvarado score below 4 in adults [18], although the authors note that absent follow-up in non-operated patients could result in under-reported cases in the low-risk group and inflated sensitivity estimates [17].

The sex distribution of the cohort does not account for the shortfall. Women formed 56.5% of the cohort but 43.3% of confirmed cases, and the systematic review on which the Alvarado comparator is based reports that the score over-predicts appendicitis in women [17]. That pattern is reproduced here in specificity, which was 0.841 in women against 0.909 in men, but it does not carry the sensitivity finding, since sensitivity at the Alvarado threshold did not differ between sexes (*p* = 0.306) and the shortfall was present in both sexes for all three instruments.

Restricting the analysis to patients meeting those entry criteria was considered and not adopted. The source cohorts entered patients on clinical suspicion of appendicitis, a judgement formed before any score is computed; no equivalent judgement is recorded here, so any subgroup available in these data must be defined by the localizing features themselves, which carry 4 of the 10 points of the Alvarado score, 4 of 12 for the AIR score and 9 to 11 of 23 for the AAS. Selecting on those features conditions on the predictor, so these estimates are reported as a sensitivity analysis rather than a primary result (Supplementary Table S6). Among the 240 patients with at least one localizing feature, where prevalence is 56.7% and therefore within the 32% to 91% range of the source studies, sensitivity at the recommended thresholds was 0.824 for the Alvarado score, 0.985 for the AIR score and 0.912 for the AAS. Restriction changes the picture differently for each instrument: the Alvarado threshold remains 17 percentage points below its pooled value, the AAS closes to within 5 points, and the AIR threshold reaches 0.985 against a pooled 0.95. The same instrument at the same cut point therefore moves by 14 to 22 percentage points on a change in case mix alone.

### 4.3. Discrimination, Calibration and Clinical Utility

Discrimination was preserved within the low-risk band. Among patients below each rule-out threshold, where every patient receives the same recommendation, the scores retained an area under the curve of 0.697 to 0.739, so dichotomization at the recommended cut point discards information the score still carries. At the opposite end, 65 of the 84 patients with an Alvarado score of 7 or above had confirmed appendicitis, and the rule-in direction therefore performed well in the same cohort in which the rule-out direction failed. When thresholds were re-derived within this cohort at a matched safety target, the scores avoided CT in 162 to 221 patients while missing between two and five cases. What does not transfer is the numerical cut point rather than the instrument. The model’s behaviour points the same way: its deferrals fell almost entirely within the scores’ own low-risk bands, so it identified the same low-risk region and differed only in where it placed the boundary. Physical examination, represented here by a single binary composite of right lower quadrant tenderness, guarding or rebound tenderness, contributed a small increment to model discrimination that was not distinguishable from zero once the fitting procedure was resampled. Its magnitude is consistent with the meta-analytic finding that individual clinical and laboratory variables are weak discriminators in isolation and achieve useful accuracy only in combination [26]; this is a lower bound on what a graded examination might contribute.

Adding examination and laboratory information to a symptom-only model raised discrimination from 0.874 to 0.918, whereas the addition of structured CT findings raised it to 0.995, exceeding the two pre-imaging increments combined, and radiologist interpretation added nothing measurable beyond the structured CT variables; the same pattern held with a single algorithm fixed across layers. Existing machine learning work on appendicitis has been evaluated as classification against a reference standard, in undifferentiated emergency populations [12], in patients preselected for equivocal CT findings [13], and in operated cohorts with severity as the outcome [14]. The quantity relevant to imaging policy, namely the proportion of examinations that could have been withheld at a stated safety level, cannot be recovered from a discrimination statistic alone.

### 4.4. Clinical Implications

The clinical implications concern the patient with acute abdominal pain for whom imaging is being considered, not the patient in whom appendicitis has already been diagnosed. The scores are applied at exactly this point, before the diagnosis is known, and a score at or below the recommended threshold is taken as grounds for withholding imaging. In this cohort, that rule would have withheld CT from 57, 27 and 54 patients with confirmed appendicitis. The finding does not argue against the scores, which discriminated as expected, nor does it establish a new threshold for clinical use. It indicates that a rule-out cut point derived in patients selected for suspected appendicitis should not be assumed to hold in the broader population in which the imaging decision is made, and that local verification should precede its use for that purpose. In practical terms, the published rule-out thresholds should not be applied unchanged to defer CT in patients in whom imaging is already being considered; a threshold intended for that purpose should first be re-derived and verified in the population in which it is to be used.

### 4.5. Limitations

The study has several limitations. The cohort was defined by the performance of CT. All eligible patients who underwent CT for acute abdominal pain were included by design, without restriction to suspected appendicitis, so that the alternative causes of acute abdominal pain were represented, and no selection was applied among imaged patients. The decision to image was nevertheless made by the treating physician without a protocol, so patients in whom imaging was judged unnecessary are absent, and the cohort represents those in whom diagnostic uncertainty was sufficient to prompt CT rather than all patients with acute abdominal pain. This is the population in which a rule-out threshold would be applied to defer imaging, but the findings may not generalise to settings with different imaging practice. Characteristics of the 333 excluded patients were not extracted, so the possibility that exclusion for incomplete laboratory data was related to clinical presentation cannot be assessed. Right lower quadrant tenderness, rebound tenderness and guarding were recorded as a single binary finding without severity grading, so the graded components of the AIR score and the AAS were assigned rather than measured; the assignment used was the one most favorable to the scores, and the full range of alternatives is reported in Supplementary Table S1. Score components were reconstructed from routine documentation, and absence in the record was treated as absence of the finding, so the possibility that some localizing findings were present but undocumented cannot be excluded. Verification was differential. An appendicitis that resolved without operation or that was not identified on the index CT would have been classified as absent, and no structured follow-up window was available to detect such cases. The direction of this misclassification is conservative with respect to the primary finding, since any unrecognized case falling within a low probability stratum would further reduce the sensitivity of the corresponding rule-out threshold rather than increase it. Discrimination at M4 and M5 is subject to incorporation bias: 571 of the 583 patients classified as free of appendicitis carried a radiologist reading of no appendicitis, and that reading enters at M5; thus, the increment from M3 to M4 measures the ceiling imposed by a CT-based reference standard rather than independent predictive information. Re-estimating it in the 180 operated patients, in whom the reference standard is histopathology and independent of the CT, is not possible, because only two of them were free of appendicitis. Three of the 31 predictors at M3 are deterministic dichotomizations of three others and carry no information the continuous variables lack for a tree ensemble; removing them would raise the events-per-variable ratio from 5.7 to 6.4. Model thresholds were derived and evaluated within a single cohort by cross-validation, so external validation was not performed. The model analysis is therefore exploratory, and neither the models nor the re-derived score thresholds are proposed for clinical use; the reported numbers of CT examinations avoided are internal estimates that require confirmation in an independent cohort. In a temporal split of the cohort, the shortfall of the recommended thresholds was present in both periods, but the M3 threshold derived in the earlier period attained a sensitivity of 0.925 (0.837 to 0.968) in the later one, a point estimate below the clinical target, so the model threshold lost sensitivity when transferred across time. The algorithm carried forward at each layer was the one with the highest cross-validated AUC among eleven candidates, and that maximum is reported as the layer’s performance; selecting the best of eleven correlated estimates is optimistic, and the clinical scores carry no equivalent selection step. The spread across algorithms within a layer was narrow, with the highest exceeding the median by 0.014 at M1, 0.016 at M2 and 0.011 at M3 (Supplementary Table S7). Thus, the magnitude is small, and the model-versus-score contrasts should be read with this in mind. With the selection nested inside the cross-validation, the area under the curve of M3 was 0.913 against 0.918, and its contrast against the AAS was no longer distinguishable from zero (Supplementary Table S4). The ratio of outcome events to candidate predictors also falls below the conventional floor at the later layers, at 5.7 for M3 and 4.5 for M5. M3 is the model relevant to the pre-imaging decision, and at this ratio its estimates are less stable than the confidence intervals alone suggest; the calibration slope of 1.27 (95% CI 1.08 to 1.46), indicating predictions compressed towards the cohort mean, is the pattern of an averaged tree ensemble, and the low event-per-variable ratio adds instability to it. Histopathological severity was not recorded in the source dataset, so the missed cases could not be classified as uncomplicated, gangrenous or perforated. The sample size was fixed by data availability rather than by a formal calculation.

The study was conducted at a tertiary university hospital, where the case mix differs from that of the populations in which the scores were derived. Women comprised 56.5% of the cohort but 43.3% of confirmed cases, and the median age was 48 years, higher than in most published appendicitis cohorts. Fever was uncommon, with a temperature of 37.3 degrees Celsius or above recorded in 14.1% of the cohort and 22.5% of confirmed cases, and a temperature of 38.5 degrees Celsius or above, the threshold used by the AIR score, in only 3.9% of confirmed cases. The negative appendectomy rate was 1.1% (2 of 180), lower than is typically reported in adult series. This is the expected consequence of a pathway in which every operated patient had undergone CT before the decision to operate, with preoperative imaging being the established mechanism by which negative appendectomy rates fall. It also confirms that this is a setting in which imaging is used liberally and therefore one in which a reduction in CT use would have its largest effect. Referral patterns to a tertiary center may favor atypical or diagnostically difficult presentations, and the extent to which these findings apply to secondary care or to unselected emergency populations requires separate evaluation. Data were collected over more than ten years, from November 2015 to April 2026, during which imaging practice, CT protocols and laboratory methods may have changed. The prevalence of appendicitis was 26.6% before 2024 and 19.5% afterwards; in the later period, discrimination was lower than in the cross-validated analysis (0.883 against 0.918 for M3) and over-prediction larger, whereas the shortfall of the recommended thresholds was present in both periods (Table 5).

## 5. Conclusions

In a population defined by imaging rather than by clinical suspicion, the recommended rule-out thresholds of the Alvarado score, the AIR score and the AAS did not translate, most clearly for the Alvarado score. The instruments discriminated as expected, and the failure was specific to the transferred cut point and concentrated among early presentations in which pain had not yet localized. Any policy that defers imaging based on a recommended threshold should first establish that the threshold was derived in a population resembling the one in which it is to be applied. These findings come from a single centre without external validation and should be confirmed in independent cohorts before they inform practice.

## Figures and Tables

**Table 1:** Baseline characteristics of the analysis cohort (*n* = 761).

Characteristic	All (n = 761)	Appendicitis Present (n = 178)	Appendicitis Absent (n = 583)	*p*-Value
**Continuous variables, median (range)**				
***L1—Clinical symptoms and demographics***				
** Age, years**	48 (16–95)	40 (16–89)	50 (17–95)	**<0.001**
** Body temperature, °C**	36.7 (35.9–39.6)	36.7 (35.9–39.4)	36.6 (35.9–39.6)	**0.047**
** Pain onset to presentation, h**	20 (1–720)	23 (1–504)	18 (1–720)	0.794
***L3—Laboratory***				
** White blood cell count, ×10^9^/L**	10.2 (2.3–35.3)	13.7 (3.0–28.9)	9.5 (2.3–35.3)	**<0.001**
** Absolute neutrophil count, ×10^9^/L**	7.1 (0.3–31.5)	11.1 (1.7–23.7)	6.3 (0.3–31.5)	**<0.001**
** Absolute lymphocyte count, ×10^9^/L**	1.8 (0.2–8.8)	1.5 (0.4–5.7)	1.9 (0.2–8.8)	**<0.001**
** Neutrophil percentage, %**	72.0 (8.4–98.0)	80.0 (51.0–98.0)	68.0 (8.4–98.0)	**<0.001**
** Lymphocyte percentage, %**	18.0 (1.0–53.0)	12.0 (2.3–38.0)	21.0 (1.0–53.0)	**<0.001**
** C-reactive protein, mg/L**	7.2 (0.3–535.9)	16.4 (0.6–509.0)	5.5 (0.3–535.9)	**<0.001**
** Haemoglobin, g/dL**	13.1 (4.3–17.7)	13.9 (9.8–17.4)	12.9 (4.3–17.7)	**<0.001**
** Serum potassium, mmol/L**	4.2 (2.9–5.8)	4.2 (3.1–5.4)	4.2 (2.9–5.8)	0.197
** Serum sodium, mmol/L**	138.0 (105.0–152.0)	138.0 (130.0–148.0)	139.0 (105.0–152.0)	**<0.001**
***L4—Structured CT findings***				
** Appendiceal diameter, mm**	5 (2–27)	11 (5–27)	5 (2–13)	**<0.001**
** *Scores***				
** Alvarado score (0–10)**	3 (0–10)	6 (0–10)	2 (0–9)	**<0.001**
** AIR score (0–12)**	3 (0–11)	6 (0–11)	2 (0–10)	**<0.001**
** Adult Appendicitis Score (0–23)**	6 (0–23)	14 (2–23)	5 (0–19)	**<0.001**
** Categorical variables, n (%)**				
***L1—Clinical symptoms and demographics***				
** Female sex**	430 (56.5%)	77 (43.3%)	353 (60.5%)	**<0.001**
** ** **Fever (documented, ≥ 37.8 °C)**	60 (7.9%)	23 (12.9%)	37 (6.3%)	**0.007**
** Body temperature ≥ 37.3 °C**	107 (14.1%)	40 (22.5%)	67 (11.5%)	**<0.001**
** Nausea or vomiting**	250 (32.9%)	67 (37.6%)	183 (31.4%)	0.143
** Anorexia**	111 (14.6%)	44 (24.7%)	67 (11.5%)	**<0.001**
** Pain migration**	81 (10.6%)	51 (28.7%)	30 (5.1%)	**<0.001**
** Right lower quadrant pain**	180 (23.7%)	103 (57.9%)	77 (13.2%)	**<0.001**
***L2—Physical examination***				
** RLQ tenderness, guarding, or rebound**	218 (28.6%)	131 (73.6%)	87 (14.9%)	**<0.001**
***L3—Laboratory***				
** Leukocytosis (WBC > 10 ×10^9^/L)**	392 (51.5%)	133 (74.7%)	259 (44.4%)	**<0.001**
** Neutrophilia (neutrophil percentage ≥**75%)	337 (44.3%)	128 (71.9%)	209 (35.8%)	**<0.001**
** Elevated C-reactive protein (> 5 mg/L)**	429 (56.4%)	129 (72.5%)	300 (51.5%)	**<0.001**
***L4—Structured CT findings***				
** Appendiceal wall enhancement**	167 (21.9%)	162 (91.0%)	5 (0.9%)	**<0.001**
** Periappendiceal fat stranding**	168 (22.1%)	161 (90.4%)	7 (1.2%)	**<0.001**
** Intra-abdominal free air**	24 (3.2%)	18 (10.1%)	6 (1.0%)	**<0.001**
** Free peritoneal fluid**	127 (16.7%)	42 (23.6%)	85 (14.6%)	**0.007**
** Mesenteric lymphadenopathy**	60 (7.9%)	34 (19.1%)	26 (4.5%)	**<0.001**
***L5—Radiologist interpretation***				
**CT comment**				**<0.001**
** No appendicitis**	574 (75.4%)	3 (1.7%)	571 (97.9%)	
** Possible appendicitis**	80 (10.5%)	69 (38.8%)	11 (1.9%)	
** Appendicitis**	107 (14.1%)	106 (59.6%)	1 (0.2%)	

AIR: Appendicitis Inflammatory Response; CT: computed tomography; RLQ: right lower quadrant; WBC: white blood cell count. Continuous variables are presented as median (minimum–maximum) and categorical variables as n (%). Significant *p*-values (<0.05) are shown in bold. Continuous variables were compared using the Mann–Whitney U test; categorical variables using the chi-squared test, or Fisher’s exact test where any cell count was below five, and the three-level radiologist interpretation using a chi-squared test across all categories.

**Table 2:** Clinical score distribution, discrimination and performance of recommended rule-out thresholds (*n* = 761).

**A. Score distribution and discrimination across the full cohort**							
**Score**	**Theoretical range**	**Observed range**	**Median (IQR), all**	**Median (IQR), AP**	**Median (IQR), no AP**		**AUC (95% CI)**
**Alvarado**	0–10	0–10	3 (1 to 5)	6 (4 to 7)	2 (0 to 4)		0.848 (0.815 to 0.881)
**AIR**	0–12	0–11	3 (1 to 5)	6 (5 to 8)	2 (1 to 4)		0.868 (0.837 to 0.896)
**AAS**	0–23	0–23	6 (3 to 10)	14 (9 to 17)	5 (3 to 7)		0.881 (0.850 to 0.910)
**B. Performance at the recommended rule-out threshold**							
**Score**	**Threshold**	**Low-risk band, n (%)**	**Confirmed AP in band, n (%)**	**Sensitivity (95% CI)**	**Specificity (95% CI)**	**NPV (95% CI)**	AUC within band (95% CI) *
**Alvarado**	≤4	563 (74.0)	57 (10.1)	0.680 (0.609 to 0.751)	0.868 (0.841 to 0.895)	0.899 (0.872 to 0.923)	0.697 (0.637 to 0.757)
**AIR**	≤3	430 (56.5)	27 (6.3)	0.848 (0.791 to 0.899)	0.691 (0.654 to 0.729)	0.937 (0.912 to 0.959)	0.726 (0.652 to 0.795)
**AAS**	≤10	582 (76.5)	54 (9.3)	0.697 (0.628 to 0.764)	0.906 (0.881 to 0.929)	0.907 (0.882 to 0.930)	0.739 (0.676 to 0.801)

AP, acute appendicitis; AIR, Appendicitis Inflammatory Response; AAS, Adult Appendicitis Score; AUC, area under the receiver operating characteristic curve; IQR, interquartile range; NPV, negative predictive value. * Rule-out thresholds are the lowest risk stratum recommended for each instrument. For the Alvarado score and the AAS, these are the strata defined in the source literature. For the AIR score, 3 or below is applied here as the more conservative comparator, with results at its original cut-off of 4 or below reported in Supplementary Table S2. AUC within band was calculated among patients below the recommended rule-out threshold only and quantifies the discriminative information discarded by dichotomisation.

**Table 3:** Performance of the recommended rule-out thresholds and of the M3 deferral rule by sex (*n* = 761).

Instrument	Threshold	Sensitivity, Women (n = 430, 77 AP)	Sensitivity, Men (n = 331, 101 AP)	*p*	Specificity, Women	Specificity, Men	*p*
**Alvarado**	≤4	0.727 (0.623 to 0.824)	0.644 (0.549 to 0.736)	0.306	0.841 (0.803 to 0.878)	0.909 (0.871 to 0.945)	0.026
**AIR**	≤3	0.831 (0.746 to 0.909)	0.861 (0.792 to 0.925)	0.729	0.674 (0.624 to 0.721)	0.717 (0.658 to 0.775)	0.312
**AAS**	≤10	0.675 (0.571 to 0.776)	0.713 (0.620 to 0.803)	0.707	0.907 (0.876 to 0.937)	0.904 (0.865 to 0.942)	1.000
**M3 (fold-external threshold)**	fitted at ≥ 0.97	0.961 (0.892 to 0.987)	0.970 (0.916 to 0.990)	1.000	0.555 (0.504 to 0.606)	0.509 (0.446 to 0.575)	0.309

AP: acute appendicitis.

**Table 4:** Confirmed appendicitis without (*n* = 42) and with (*n* = 136) a localizing feature.

Characteristic	No Localizing Feature (n = 42)	Other Confirmed Cases (n = 136)	*p* Value
**Demographics and symptoms**			
Age, years	38 (22–76)	41 (16–89)	0.381
Female sex	20 (47.6%)	57 (41.9%)	0.635
Pain onset to presentation, h	12 (1–160)	24 (2–504)	0.022
Nausea or vomiting	21 (50.0%)	46 (33.8%)	0.087
Anorexia	13 (31.0%)	31 (22.8%)	0.386
Body temperature, °C	36.5 (35.9–38.3)	36.8 (36.0–39.4)	0.006
Fever (documented, ≥37.8 °C)	3 (7.1%)	20 (14.7%)	0.293
Body temperature ≥ 37.3 °C	4 (9.5%)	36 (26.5%)	0.021
Body temperature ≥ 38.5 °C	0 (0.0%)	7 (5.1%)	0.201
**Localizing features**			
Right lower quadrant pain	0 (0.0%)	103 (75.7%)	—
RLQ tenderness, guarding, or rebound	0 (0.0%)	131 (96.3%)	—
Pain migration	0 (0.0%)	51 (37.5%)	—
**Laboratory**			
White blood cell count, ×10^9^/L	14.1 (5.4–28.9)	13.5 (3.0–26.7)	0.451
Absolute neutrophil count, ×10^9^/L	11.5 (3.9–23.1)	11.0 (1.7–23.7)	0.623
Absolute lymphocyte count, ×10^9^/L	1.7 (0.8–5.7)	1.5 (0.4–3.6)	0.176
Neutrophil percentage, %	80.0 (53.0–98.0)	79.5 (51.0–95.0)	0.794
Lymphocyte percentage, %	12.0 (2.3–38.0)	12.0 (3.0–36.0)	0.696
C-reactive protein, mg/L	7.9 (0.6–268.0)	21.4 (0.6–509.0)	0.009
Leukocytosis (WBC > 10 ×10^9^/L)	32 (76.2%)	101 (74.3%)	0.962
Neutrophilia (neutrophil percentage ≥ 75%)	30 (71.4%)	98 (72.1%)	1.000
Elevated C-reactive protein (>5 mg/L)	28 (66.7%)	101 (74.3%)	0.444
**Structured CT findings**			
Appendiceal diameter, mm	11 (5–18)	11 (5–27)	0.617
Appendiceal wall enhancement	39 (92.9%)	123 (90.4%)	0.766
Periappendiceal fat stranding	38 (90.5%)	123 (90.4%)	1.000
Intra-abdominal free air	1 (2.4%)	17 (12.5%)	0.077
Free peritoneal fluid	9 (21.4%)	33 (24.3%)	0.865
Mesenteric lymphadenopathy	10 (23.8%)	24 (17.6%)	0.507
Radiologist interpretation			0.843
No appendicitis	1 (2.4%)	2 (1.5%)	
Possible appendicitis	15 (35.7%)	54 (39.7%)	
Appendicitis	26 (61.9%)	80 (58.8%)	
**Clinical scores and model**			
Alvarado score	3 (0–6)	6 (0–10)	<0.001
AIR score	3 (0–6)	7 (2–11)	<0.001
AAS score	7 (2–10)	15 (6–23)	<0.001
Inside Alvarado low-probability stratum (≤4)	33 (78.6%)	24 (17.6%)	<0.001
Inside AIR low-probability stratum (≤3)	25 (59.5%)	2 (1.5%)	<0.001
Inside AAS low-probability stratum (≤10)	42 (100.0%)	12 (8.8%)	<0.001
Below the M3 deferral threshold (CT would have been deferred)	6 (14.3%)	0 (0.0%)	<0.001

Localizing features: right lower quadrant pain; tenderness, guarding or rebound; pain migration. Values are median (range) or n (%); Mann–Whitney U, Fisher’s exact or chi-square test. AAS, Adult Appendicitis Score; AIR, Appendicitis Inflammatory Response; CT, computed tomography; RLQ, right lower quadrant; WBC, white blood cell count.

**Table 5:** Temporal validation, with development before 1 January 2024 and tests from that date (*n* = 761, 178 confirmed appendicitis).

**A. Periods**					
**Period**	**Presentation dates**			**Patients, *n***	**Appendicitis, *n* (%)**
**Development**	November 2015 to December 2023			418	111 (26.6)
**Test**	January 2024 to April 2026			343	67 (19.5)
**B. Discrimination and calibration in the test period**					
**Model**	**Algorithm Selected in Development**	**AUC, Development (Cross-Validated)**	**AUC, Test (95% CI)**	**Calibration Slope, Test (95% CI)**	**Calibration Intercept, Test (95% CI)**
**M1**	Random forest	0.895	0.836 (0.788 to 0.880)	0.88 (0.65 to 1.12)	−0.99 (−1.31 to −0.67)
**M2**	Random forest	0.909	0.854 (0.807 to 0.896)	0.91 (0.68 to 1.14)	−1.06 (−1.39 to −0.74)
**M3**	Random forest	0.945	0.883 (0.832 to 0.927)	1.12 (0.85 to 1.39)	−0.72 (−1.04 to −0.39)
**C. CT deferral in the test period with thresholds re-derived in the development period**					
**Instrument**	**Threshold (Development)**		**CT Deferred, n (%) of 343**	**Missed, n of 67**	**Sensitivity (95% CI)**
**Alvarado**	≤0		77 (22.4)	1	0.985 (0.920 to 0.997)
**AIR**	≤1		94 (27.4)	1	0.985 (0.920 to 0.997)
**AAS**	≤2		65 (19.0)	1	0.985 (0.920 to 0.997)
**M3**	predicted risk < 0.165		163 (47.5)	5	0.925 (0.837 to 0.968)
**D. Recommended rule-out thresholds in each period**					
**Instrument**	**Threshold**	**Missed, Development (of 111)**	**Sensitivity, Development (95% CI)**	**Missed, Test (of 67)**	**Sensitivity, Test (95% CI)**
**Alvarado**	≤4	34	0.694 (0.608 to 0.780)	23	0.657 (0.537 to 0.769)
**AIR**	≤3	18	0.838 (0.769 to 0.905)	9	0.866 (0.779 to 0.940)
**AAS**	≤10	35	0.685 (0.600 to 0.769)	19	0.716 (0.603 to 0.819)

The best development algorithm, selected by 10-fold cross-validation, was refitted on all development patients and applied once to the test period. Thresholds in C are the highest, giving a development sensitivity of 0.97 or above; 95% CI by percentile bootstrap with 3000 resamples for the AUC and D, Wald for calibration and Wilson for C. AAS, Adult Appendicitis Score; AIR, Appendicitis Inflammatory Response; AUC, area under the receiver operating characteristic curve.

## Data Availability

The datasets generated during the current study are not publicly available due to privacy and ethical restrictions but are available from the corresponding author on reasonable request.

## Supplementary Materials

The following supporting information can be downloaded at https://www.mdpi.com/article/10.3390/jcm15208141/s1, Figure S1: Study design and analytical workflow; Figure S2: Calibration of out-of-fold predictions; Table S1: Sensitivity under every alternative grade assignment; Table S2: AIR score at its original rule-out threshold; Table S3: Algorithms and hyperparameters; Table S4: Nested cross-validation; Table S5: Performance in the held-out test set; Table S6: Performance in the indicated subgroup; Table S7: Cross-validated AUC of the eleven algorithms at each cumulative feature set; Table S8: Ridge logistic regression held fixed across layers, single layers and laboratory-first order.

## Abbreviations

The following abbreviations are used in this manuscript:

AAS	Adult Appendicitis Score
AIR	Appendicitis Inflammatory Response
AUC	Area under the receiver operating characteristic curve
CI	Confidence interval
CRP	C-reactive protein
CT	Computed tomography
ED	Emergency department
IQR	Interquartile range
L1–L5	Information layers (L1, patient-reported symptoms and demographics; L2, physical examination findings; L3, routine laboratory results; L4, structured CT findings; L5, radiologist’s final CT interpretation)
M1–M5	Cumulative feature sets corresponding to information layers L1 to L5
NPV	Negative predictive value
TRIPOD+AI	Transparent Reporting of a multivariable prediction model for Individual Prognosis Or Diagnosis, updated for artificial intelligence
