# Supplementary material for: Clinical Scores and Machine Learning at Matched Variable Domains in Acute Appendicitis: Performance of Recommended Rule-Out Thresholds in a CT-Verified Cohort
Source: J Clin Med. 2026 Oct 20;15(20):8141. doi: 10.3390/jcm15208141 (PMC13705786; doi:10.3390/jcm15208141)
Supplement: Supplementary file 1 [file jcm-15-08141-s001.zip › jcm-4590279-supplementary.pdf]

Supplementary Material

Clinical scores and machine learning at matched variable domains in acute appendicitis: performance of recommended rule-out thresholds in a CT-verified cohort

Contents

- Supplementary Figure S1. Study design and analytical workflow.
- Supplementary Figure S2. Calibration of out-of-fold predictions.
- Supplementary Table S1. Sensitivity under every alternative grade assignment.
- Supplementary Table S2. AIR score at its original rule-out threshold.
- Supplementary Table S3. Algorithms and hyperparameters.
- Supplementary Table S4. Nested cross-validation.
- Supplementary Table S5. Performance in the held-out test set.
- Supplementary Table S6. Performance in the indicated subgroup.
- Supplementary Table S7. Cross-validated AUC of the eleven algorithms at each cumulative feature set.
- Supplementary Table S8. Ridge logistic regression held fixed across layers, single layers and laboratory-first order.

Supplementary Figure S1. Study design and analytical workflow.

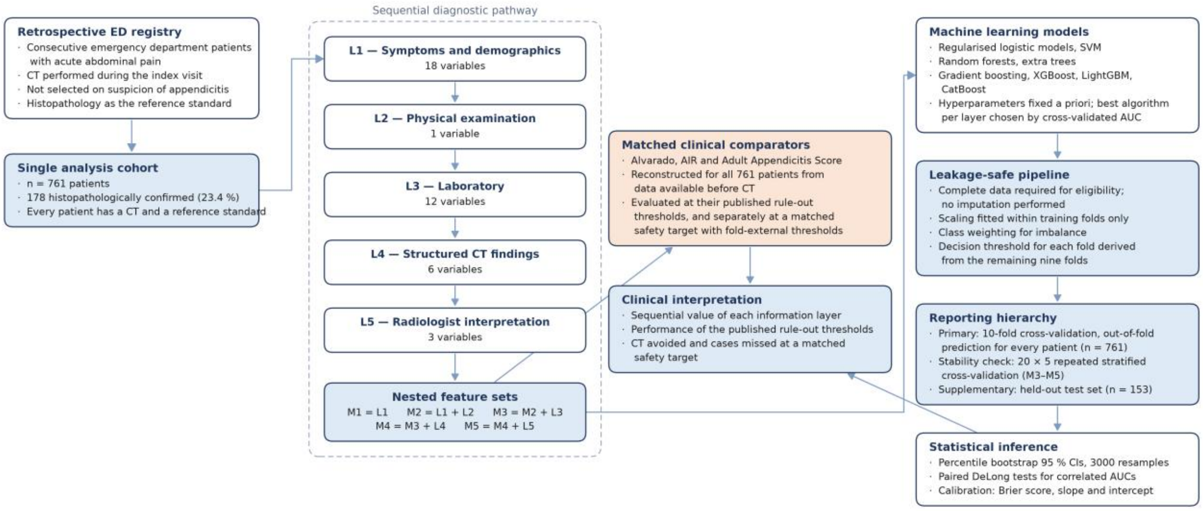

A single cohort of 761 patients was derived from a retrospective emergency department registry; patients were included on the basis of having undergone CT, not on suspicion of appendicitis. Predictors were organized into five sequential diagnostic information layers and evaluated through cumulative feature sets from M1 to M5. All estimates reported in the main text are out-of-fold, from 10-fold stratified cross-validation over the whole cohort.

Supplementary Figure S2. Calibration of out-of-fold predictions (M1 to M5).

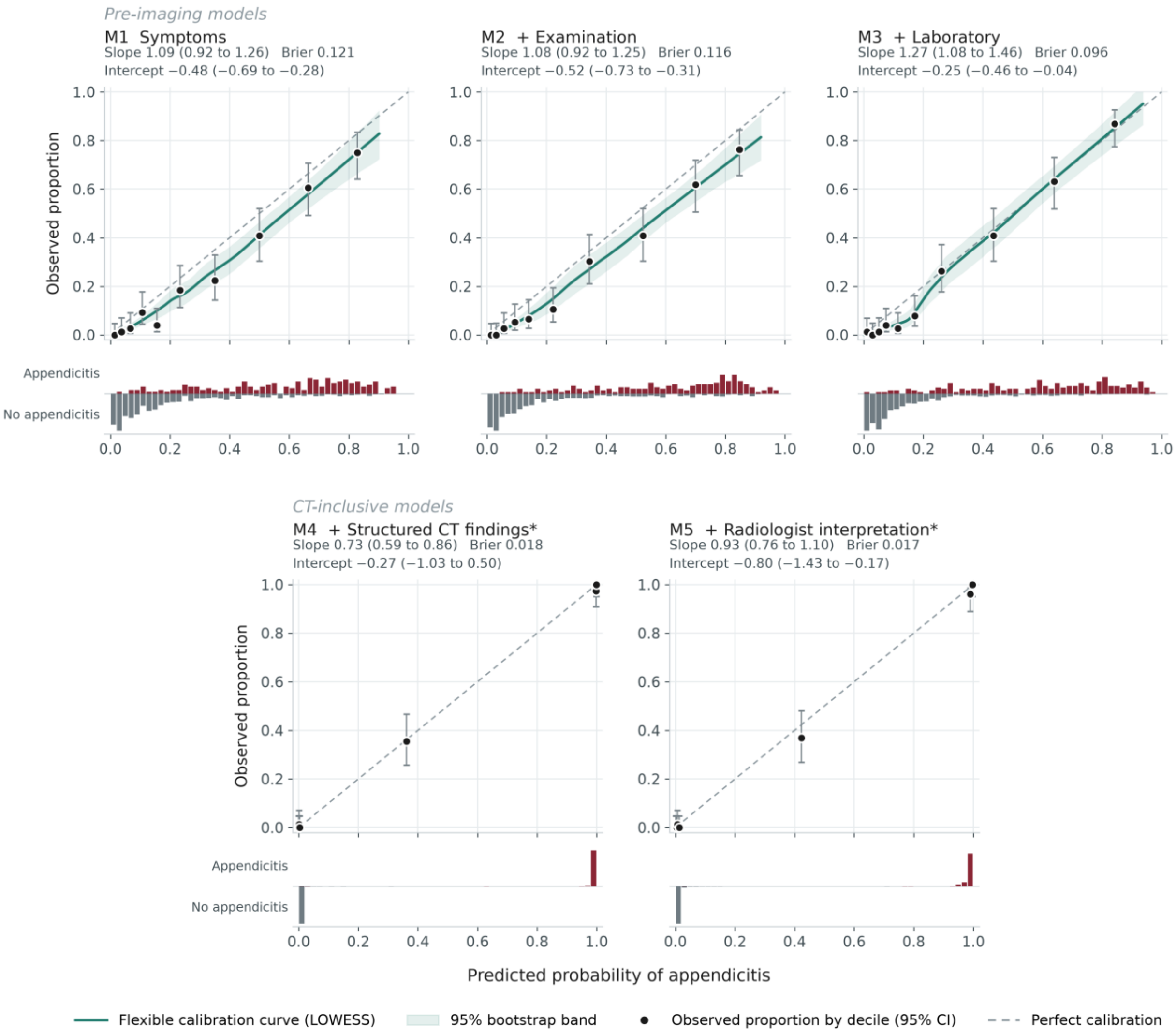

Calibration curves were drawn by locally weighted scatterplot smoothing of the observed outcome on the predicted probability, with a 95% bootstrap band, and observed proportions within deciles of predicted risk shown with 95% confidence intervals. Bars under each panel show the percentage of patients with (upward) and without (downward) appendicitis at each predicted probability. *Nearly all predictions at M4 and M5 lie near 0 or 1, so only grouped proportions are shown for these layers.

Supplementary Table S1. Sensitivity at the recommended rule-out threshold under every alternative grade assignment for the ungraded examination finding (n = 761, 178 confirmed appendicitis).

**Score**	**Threshold**	**Grade assigned to the examination finding**	**Low-risk band, n (%)**	**Cases in band**	**Sensitivity (95% CI)**	**Specificity**	**NPV**
AIR	≤ 3	light (1 point)	487 (64.0%)	44	0.753 (0.686 to 0.815)	0.760	0.910
AIR	≤ 3	medium (2 points)	456 (59.9%)	35	0.803 (0.741 to 0.861)	0.722	0.923
AIR	≤ 3	strong (3 points)	430 (56.5%)	27	0.848 (0.791 to 0.899)	0.691	0.937
AAS	≤ 10	mild (2 points)	616 (80.9%)	69	0.612 (0.541 to 0.686)	0.938	0.888
AAS	≤ 10	moderate or severe (4 points)	582 (76.5%)	54	0.697 (0.628 to 0.764)	0.906	0.907
Alvarado	≤ 4	not graded	563 (74.0%)	57	0.680 (0.609 to 0.751)	0.868	0.899

Right lower quadrant tenderness, guarding and rebound tenderness were documented as a single composite binary finding, without the severity grading required by the AIR score and the AAS. Where the finding was positive, the analysis reported in the main text assigned each instrument its highest valid grade. Assigning a lower grade lowers the score, enlarges the low-probability band and increases the number of missed cases, so the assignment used is the one most favourable to the scores. The Alvarado score has no graded examination item; both of its examination items were assigned from the composite finding. All confidence intervals are percentile intervals from 3000 patient-level bootstrap resamples with the seed fixed at 42, the same procedure used in Table 2B. NPV, negative predictive value.

Supplementary Table S2. Performance of the AIR score at its original rule-out threshold of 4 or below, alongside the threshold of 3 or below used in the main analysis (n = 761, 178 confirmed appendicitis).

Threshold	Pooled sensitivity reported	Low-risk band, n (%)	Cases in band	Sensitivity (95% CI)	Specificity	NPV
≤ 3	0.95	430 (56.5%)	27	0.848 (0.791 to 0.899)	0.691	0.937
≤ 4	0.91	523 (68.7%)	42	0.764 (0.699 to 0.825)	0.825	0.920

The AIR score was published with a low-probability stratum of 0 to 4. The subsequent meta-analysis reports a higher sensitivity at 3 or below (0.95) than at 4 or below (0.91), and 3 or below was applied in the main analysis. Both are reported here. Confidence intervals are percentile intervals from 3000 patient-level bootstrap resamples. NPV, negative predictive value.

Supplementary Table S3. Algorithms evaluated at each information layer, with the hyperparameter values fixed a priori.

Algorithm	Model family	Feature scaling	Class-imbalance handling	Hyperparameters
LogisticRegression	Regularised linear	z-score (fitted in-fold)	class_weight='balanced'	C=1.0; class_weight=balanced; dual=False; fit_intercept=True; intercept_scaling=1; max_iter=2000; multi_class=deprecated; penalty=l2; random_state=42; solver=lbfgs; tol=0.0001; verbose=0; warm_start=False
ElasticNetLogistic	Regularised linear	z-score (fitted in-fold)	class_weight='balanced'	C=0.5; class_weight=balanced; dual=False; fit_intercept=True; intercept_scaling=1; l1_ratio=0.5; max_iter=5000; multi_class=deprecated; penalty=elasticnet; random_state=42; solver=saga; tol=0.0001; verbose=0; warm_start=False
RidgeLogistic	Regularised linear	z-score (fitted in-fold)	class_weight='balanced'	C=0.25; class_weight=balanced; dual=False; fit_intercept=True; intercept_scaling=1; max_iter=5000; multi_class=deprecated; penalty=l2; random_state=42; solver=lbfgs; tol=0.0001; verbose=0; warm_start=False
SVM_RBF	Kernel SVM	z-score (fitted in-fold)	class_weight='balanced' on the inner SVC	cv=5; ensemble=auto; estimator=SVC(C=1.0, break_ties=False, cache_size=200, class_weight='balanced', coef0=0.0, decision_function_shape='ovr', degree=3, gamma='scale', kernel='rbf', max_iter=-1, probability=False, random_state=42, shrinking=True, tol=0.001, verbose=False); method=sigmoid
RandomForest	Tree ensemble (bagging)	none	class_weight='balanced'	bootstrap=True; ccp_alpha=0.0; class_weight=balanced; criterion=gini; max_features=sqrt; min_impurity_decrease=0.0; min_samples_leaf=2; min_samples_split=2; min_weight_fraction_leaf=0.0; n_estimators=500; n_jobs=-1; oob_score=False; random_state=42; verbose=0; warm_start=False
ExtraTrees	Tree ensemble (bagging)	none	class_weight='balanced'	bootstrap=False; ccp_alpha=0.0; class_weight=balanced; criterion=gini; max_features=sqrt; min_impurity_decrease=0.0; min_samples_leaf=2; min_samples_split=2; min_weight_fraction_leaf=0.0; n_estimators=500; n_jobs=-1; oob_score=False; random_state=42; verbose=0; warm_start=False
GradientBoosting	Gradient-boosted trees	none	balanced sample weights supplied at fit time	ccp_alpha=0.0; criterion=friedman_mse; learning_rate=0.05; loss=log_loss; max_depth=3; min_impurity_decrease=0.0; min_samples_leaf=1; min_samples_split=2; min_weight_fraction_leaf=0.0; n_estimators=200; random_state=42; subsample=0.8; tol=0.0001; validation_fraction=0.1; verbose=0; warm_start=False
HistGradientBoosting	Gradient-boosted trees	none	class_weight='balanced'	categorical_features=from_dtype; class_weight=balanced; early_stopping=auto; l2_regularization=0.1; learning_rate=0.05; loss=log_loss; max_bins=255; max_depth=5; max_features=1.0; max_iter=300; max_leaf_nodes=31; min_samples_leaf=20; n_iter_no_change=10; random_state=42; scoring=loss; tol=1e-07; validation_fraction=0.1; verbose=0;

				warm_start=False
XGBoost	Gradient-boosted trees	none	scale_pos_weight = negatives/positives	colsample_bytree=0.8; enable_categorical=False; eval_metric=logloss; learning_rate=0.025; max_depth=3; min_child_weight=3; missing=nan; n_estimators=400; n_jobs=-1; objective=binary:logistic; random_state=42; reg_alpha=0.1; reg_lambda=2.0; scale_pos_weight=n_negative/n_positive (computed within each training fit); subsample=0.8; verbosity=0
LightGBM	Gradient-boosted trees	none	class_weight='balanced'	boosting_type=gbdt; class_weight=balanced; colsample_bytree=0.8; importance_type=split; learning_rate=0.02; max_depth=3; min_child_samples=10; min_child_weight=0.001; min_split_gain=0.0; n_estimators=500; n_jobs=-1; num_leaves=31; random_state=42; reg_alpha=0.1; reg_lambda=2.0; subsample=0.8; subsample_for_bin=200000; subsample_freq=0; verbose=-1
CatBoost	Gradient-boosted trees	none	auto_class_weights='Balanced'	allow_writing_files=False; auto_class_weights=Balanced; depth=3; eval_metric=AUC; iterations=500; l2_leaf_reg=5.0; learning_rate=0.025; loss_function=Logloss; random_seed=42; verbose=False

Hyperparameters were fixed before any model was fitted and were not tuned by grid search. All eleven algorithms were evaluated at all five information layers. In the cross-validated primary analysis the algorithm carried forward at each layer was the one with the highest cross-validated AUC: RandomForest at M1, M2 and M3, GradientBoosting at M4 and XGBoost at M5. The held-out analysis in Supplementary Table S5 selected within its own training partition and reached a different choice at four layers. Random seeds were fixed at 42 throughout.

Supplementary Table S4. Nested cross-validation, with the selection among the eleven algorithms repeated inside the cross-validation (n = 761, 178 confirmed appendicitis).

A. Discrimination

Model	AUC, reported	AUC, nested (95% CI)	Difference	Algorithm selected (outer folds)
M1	0.874	0.862 (0.833 to 0.891)	−0.012	RandomForest 6 of 10, ExtraTrees 2 of 10, CatBoost 2 of 10
M2	0.889	0.882 (0.855 to 0.908)	−0.007	RandomForest 7 of 10, CatBoost 3 of 10
M3	0.918	0.913 (0.889 to 0.936)	−0.004	RandomForest 9 of 10, ExtraTrees 1 of 10
M4	0.995	0.994 (0.988 to 0.998)	−0.002	GradientBoosting 6 of 10, CatBoost 2 of 10, RandomForest 2 of 10
M5	0.996	0.995 (0.990 to 0.999)	−0.001	RandomForest 4 of 10, GradientBoosting 4 of 10, HistGradientBoosting 1 of 10, XGBoost 1 of 10

B. M3 versus the clinical scores

Contrast	ΔAUC, reported	P	ΔAUC, nested	P
M3 vs Alvarado	+0.065 (+0.036 to +0.093)	<0.01	+0.058 (+0.024 to +0.090)	<0.01
M3 vs AIR	+0.044 (+0.015 to +0.074)	<0.01	+0.038 (+0.006 to +0.067)	0.024
M3 vs AAS	+0.032 (+0.003 to +0.060)	0.033	+0.025 (−0.007 to +0.052)	0.114

C. CT deferral

Model	Deferred, reported	Missed	Deferred, nested	Missed	Sensitivity, nested (95% CI)
M1	264 (34.7%)	5	250 (32.9%)	6	0.966 (0.928 to 0.984)
M2	298 (39.2%)	6	287 (37.7%)	6	0.966 (0.928 to 0.984)
M3	319 (41.9%)	6	302 (39.7%)	6	0.966 (0.928 to 0.984)

Nested, algorithm selection repeated within each outer fold (A, C) or within each bootstrap resample (B; 300 resamples for the reported contrasts, 1000 for the nested contrasts). Reported, primary analysis in the main text. Difference, nested minus reported area under the curve, calculated from unrounded values. Sensitivity with Wilson 95% confidence interval.

Supplementary Table S5. Performance in the held-out test set (n = 153, 36 confirmed appendicitis).

**Feature set**	**Algorithm**	**Predictors**	**AUC (95% CI)**	**Sensitivity**	**Specificity**	**PPV**	**NPV**	**Brier score**
M1, symptoms and demographics	CatBoost	18	0.811 (0.731 to 0.880)	0.667	0.786	0.490	0.885	0.163
M2, + physical examination	HistGradientBoosting	19	0.872 (0.797 to 0.934)	0.750	0.889	0.675	0.920	0.116
M3, + laboratory	LightGBM	31	0.888 (0.822 to 0.945)	0.806	0.863	0.644	0.935	0.119
M4, + structured CT findings	GradientBoosting	37	0.992 (0.979 to 1.000)	0.833	0.992	0.968	0.951	0.030
M5, + radiologist interpretation	GradientBoosting	40	0.995 (0.984 to 1.000)	0.833	0.992	0.968	0.951	0.027

The cohort was partitioned by stratified random sampling into training (n = 456, 107 with appendicitis), validation (n = 152, 35) and held-out test (n = 153, 36) subsets. Models were fitted on the training set, the operating threshold was selected on the validation set by the Youden J statistic, and performance was measured once on the held-out test set. The algorithm carried forward at each layer was selected within this partition and therefore differs from the selection made in the cross-validated primary analysis at four of the five layers. Because the 153 held-out patients are a subset of the 761 analysed in the main text, the two analyses are not independent and these estimates are not a substitute for external validation. Discrimination is lower than in the primary cross-validated analysis, as expected when a model is fitted on 456 rather than approximately 685 patients and evaluated on 36 rather than 178 events. The operating point is the Youden J point, which maximises the sum of sensitivity and specificity and is not a rule-out threshold, so these sensitivities are not comparable with the fold-external deferral results in the main text. PPV, positive predictive value; NPV, negative predictive value.

Supplementary Table S6. Performance of the recommended rule-out thresholds under alternative definitions of the indicated subgroup.

Population	Score	Threshold	n / cases	Sensitivity (95% CI)	Cases below threshold	AUC
Full cohort	Alvarado	≤ 4	761 / 178	0.680 (0.609 to 0.751)	57	0.848
Full cohort	AIR	≤ 3	761 / 178	0.848 (0.791 to 0.899)	27	0.868
Full cohort	AAS	≤ 10	761 / 178	0.697 (0.628 to 0.764)	54	0.881
A: right lower quadrant pain	Alvarado	≤ 4	180 / 103	0.796 (0.716 to 0.870)	21	0.738
A: right lower quadrant pain	AIR	≤ 3	180 / 103	0.981 (0.950 to 1.000)	2	0.781
A: right lower quadrant pain	AAS	≤ 10	180 / 103	0.893 (0.830 to 0.949)	11	0.800
B: right lower quadrant pain or examination finding	Alvarado	≤ 4	238 / 136	0.824 (0.758 to 0.885)	24	0.715
B: right lower quadrant pain or examination finding	AIR	≤ 3	238 / 136	0.985 (0.961 to 1.000)	2	0.766
B: right lower quadrant pain or examination finding	AAS	≤ 10	238 / 136	0.912 (0.864 to 0.958)	12	0.806
C: any of the three localizing features (used)	Alvarado	≤ 4	240 / 136	0.824 (0.759 to 0.886)	24	0.720
C: any of the three localizing features (used)	AIR	≤ 3	240 / 136	0.985 (0.962 to 1.000)	2	0.770
C: any of the three localizing features (used)	AAS	≤ 10	240 / 136	0.912 (0.861 to 0.958)	12	0.810

Thresholds are those recommended for each instrument. Definitions A, B and C are nested. Definitions B and C differ by two patients, neither of whom had appendicitis, so they give identical sensitivities and identical case counts and differ only marginally in AUC; the narrower definition A gives slightly lower sensitivity for each instrument (0.796, 0.981 and 0.893). Definition C, the most inclusive, is the one referred to in the Discussion. The subgroup is defined by the localizing features themselves, which carry 4 of the 10 points of the Alvarado score, 4 of 12 for the AIR score and 9 to 11 of 23 for the AAS, so restriction on these features conditions on the predictor. Discrimination falls in the restricted subgroup for every instrument and for the model alike, which is the expected consequence of range restriction under a narrowed case mix rather than evidence about the sensitivity estimates; out-of-fold discrimination of M3 in subgroup C was 0.822, against 0.918 in the full cohort. These estimates are a sensitivity analysis and are not a primary result. All confidence intervals are percentile intervals from 3000 patient-level bootstrap resamples with the seed fixed at 42, the same procedure used in Table 2B.

Supplementary Table S7. Cross-validated AUC of the eleven algorithms at each cumulative feature set (n = 761, 178 confirmed appendicitis).

Algorithm	M1	M2	M3	M4	M5
Logistic regression	0.853	0.861	0.894	0.992	0.994
ElasticNet logistic regression	0.854	0.862	0.896	0.991	0.993
Ridge logistic regression	0.855	0.863	0.898	0.991	0.993
Support vector machine (RBF kernel)	0.834	0.851	0.906	0.981	0.986
Random forest	**0.874**	**0.889**	**0.918**	0.993	0.995
Extra trees	0.873	0.885	0.910	0.988	0.991
Gradient boosting	0.861	0.879	0.904	**0.995**	0.994
HistGradientBoosting	0.859	0.862	0.900	0.994	0.994
XGBoost	0.866	0.879	0.907	0.995	**0.996**

LightGBM	0.861	0.873	0.908	0.994	0.995
CatBoost	0.870	0.884	0.909	0.994	0.993
Mean of the eleven algorithms	0.860	0.872	0.905	0.992	0.993

Out-of-fold AUC from 10-fold stratified cross-validation. Bold, the algorithm carried forward at each layer, selected at full precision. Hyperparameters are given in Supplementary Table S3.

Supplementary Table S8. Ridge logistic regression held fixed across layers, single layers and laboratory-first order (n = 761, 178 confirmed appendicitis).

A. Cumulative feature sets

**Feature set**	**Predictors, n**	**AUC (95% CI)**	**Increment from the previous set (95% CI)**	**P**
M1	18	0.855 (0.821 to 0.886)	—	—
M2	19	0.863 (0.831 to 0.893)	+0.009 (−0.010 to +0.021)	0.267
M3	31	0.898 (0.871 to 0.924)	+0.031 (+0.004 to +0.053)	0.027
M4	37	0.991 (0.983 to 0.998)	+0.096 (+0.065 to +0.132)	<0.01
M5	40	0.993 (0.985 to 0.999)	+0.002 (−0.003 to +0.010)	0.333

B. Single layers and laboratory-first order

**Feature set**	**Predictors, n**	**AUC (95% CI)**
Single layer		
L1, symptoms and demographics (= M1)	18	0.855 (0.821 to 0.886)
L2, physical examination	1	0.793 (0.757 to 0.829)
L3, laboratory	12	0.783 (0.745 to 0.820)
Laboratory first		
L3	12	0.783 (0.745 to 0.820)
L3 + L1	30	0.890 (0.862 to 0.917)
L3 + L1 + L2 (= M3)	31	0.898 (0.871 to 0.924)

Out-of-fold AUC with percentile bootstrap 95% CI (3000 resamples). Increments and P from the patient-level bootstrap with refitting (300 resamples), so they can differ slightly from the differences between AUCs. L2 is a single binary finding, so its AUC is that of the finding itself. Ridge logistic regression used the prespecified hyperparameters in Supplementary Table S3.
